# A Joint Computational Respiratory Neural Network-Biomechanical Model for Breathing and Airway Defensive Behaviors

**DOI:** 10.3389/fphys.2012.00264

**Published:** 2012-07-23

**Authors:** Russell O’Connor, Lauren S. Segers, Kendall F. Morris, Sarah C. Nuding, Teresa Pitts, Donald C. Bolser, Paul W. Davenport, Bruce G. Lindsey

**Affiliations:** ^1^Department of Molecular Pharmacology and Physiology, Morsani College of Medicine, University of South FloridaTampa, FL, USA; ^2^Department of Physiological Sciences, College of Veterinary Medicine, University of FloridaGainesville, FL, USA

**Keywords:** biomechanical model, brainstem, breathing, chest wall dynamics, computational neural network model, cough, inspiratory drive, neuromechanical model simulation

## Abstract

Data-driven computational neural network models have been used to study mechanisms for generating the motor patterns for breathing and breathing related behaviors such as coughing. These models have commonly been evaluated in open loop conditions or with feedback of lung volume simply represented as a filtered version of phrenic motor output. Limitations of these approaches preclude assessment of the influence of mechanical properties of the musculoskeletal system and motivated development of a biomechanical model of the respiratory muscles, airway, and lungs using published measures from human subjects. Here we describe the model and some aspects of its behavior when linked to a computational brainstem respiratory network model for breathing and airway defensive behavior composed of discrete “integrate and fire” populations. The network incorporated multiple circuit paths and operations for tuning inspiratory drive suggested by prior work. Results from neuromechanical system simulations included generation of a eupneic-like breathing pattern and the observation that increased respiratory drive and operating volume result in higher peak flow rates during cough, even when the expiratory drive is unchanged, or when the expiratory abdominal pressure is unchanged. Sequential elimination of the model’s sources of inspiratory drive during cough also suggested a role for disinhibitory regulation via tonic expiratory neurons, a result that was subsequently supported by an analysis of *in vivo* data. Comparisons with antecedent models, discrepancies with experimental results, and some model limitations are noted.

## Introduction

The neural mechanisms that regulate and coordinate breathing and respiratory-related behaviors such as coughing are not well understood. This lack of knowledge hampers elucidation of pathophysiological deficits in airway protection and impedes development of new therapeutic approaches for dystussia that occur with neurological disorders (Suárez et al., 2002; McCool, 2006). Computational neural network models for breathing and the neurogenesis of cough inferred from *in vivo* experiments have iteratively aided prediction and refinement of hypotheses for further *in vivo* testing (Shannon et al., 1998, 2000; Baekey et al., 2001; Rybak et al., 2008; Poliaček et al., 2011). Such data-driven models, based in part on elements and connectivity inferred from simultaneous extracellular recordings of many brainstem neurons (e.g., Segers et al., 2008; Ott et al., 2012), have largely been evaluated in either open loop conditions or with feedback of lung volume simply represented as a filtered version of the motor output (Lindsey et al., 2012). While useful, these approaches have precluded model-based assessment of the potential influence of mechanical properties of the musculoskeletal system on respiratory motor pattern generation and cough effectiveness (Smith et al., 2012). More generally, neuromechanical models can provide a framework for estimating and predicting the extent to which motor patterns are constrained and influenced by mechanical properties and muscle synergies (Chiel et al., 2009).

A model that relates a respiratory neural output to mechanical outputs has been available for some time (Riddle and Younes, 1981; Younes and Riddle, 1981; Younes et al., 1981) and remains an important element in contemporary models of the respiratory system (Cheng et al., 2010; Cheng and Khoo, 2012). However, the Younes–Riddle model with its single inspiratory neural output and single state variable (lung volume) lacks features essential for model-based assessment of the respective contributions and interactions of neural and biomechanical mechanisms during cough. We have developed a respiratory neural network model with inspiratory (phrenic), expiratory (lumbar), and laryngeal neural outputs and required a mechanical model with corresponding inputs to control the abdominal, diaphragm, and laryngeal muscles. Moreover, it is well known that a given lung volume can be achieved with different configurations of the rib cage and abdomen (Konno and Mead, 1967; Younes and Riddle, 1981), and with separate neural control of the diaphragm and abdominal wall muscles as in our network model, all of these configurations can potentially be achieved. Thus, the first aim of the work reported here was to develop a model of the mechanical respiratory system that includes separate muscle models for the diaphragm, abdominal wall, and larynx, and two state variables to represent the thoracoabdominal configuration.

Our second aim was to link the resulting mechanical subsystem to an enhanced integrate and fire (IF) neural network model of the brainstem network for respiratory motor pattern generation and to assess the integrated system’s behavior with muscle activation parameters for eupneic conditions. A third related objective was to extend the simulations to include evoked coughs in order to evaluate the model’s performance in response to defined perturbations that enhance or reduce inspiratory drive. This latter goal was motivated in part by evidence that changes in the inspiratory or “operating” volume can influence airflow during the expulsive phase of the cough (Smith et al., 2012).

An additional impetus for the third aim came from results of recent model simulations, which suggested that elevated systemic arterial blood pressure – such as may occur during coughing (Sharpey-Schafer, 1953) – attenuates cough inspiratory drive, a result supported by coordinated *in vivo* experiments (Poliaček et al., 2011). The network model used in the present work builds upon that and other recent prior efforts (Rybak et al., 2008). The network incorporates multiple circuit paths and operations for tuning inspiratory drive that have been inferred from spike train cross-correlation feature sets (Lindsey et al., 1998; Shannon et al., 2000; Segers et al., 2008; Ott et al., 2012). These circuits include parallel channels for modulation of inspiratory phase activity in “tonic” expiratory neurons that inhibit premotor inspiratory bulbospinal neurons and drive.

In the course of sequentially eliminating sources of inspiratory drive for cough in the neuromechanical model, we also noted a contribution of tonic expiratory neuron activity to modulation of inspiratory phase drive during cough. This disinhibitory regulation predicted from the modeling results was subsequently supported by an analysis of *in vivo* data as described in a companion report (Segers et al., 2012).

## Materials and Methods

Neural circuit components were derived from previously described respiratory network models of discrete “IF” populations after MacGregor (1987) and a “hybrid IF burster” population with Hodgkin–Huxley style equations after Breen et al. (2003). These models were developed iteratively with *in vivo* experiments that both guided model development and tested model predictions, as detailed in Rybak et al. (2008) and Poliaček et al. (2011). The enhanced network model used herein is described further in the Results.

Biomechanical model elements were developed using parameters derived from published work as described in Results. Of particular importance was the work of Grassino et al. (1978), who measured transdiaphragmatic pressure and diaphragm activation while controlling the thoracoabdominal configuration, making it possible to estimate the effect of rib cage motion on the abdominal volume. Our abdominal wall model is based on measurements of the curvature of the abdomen by Song et al. (2006) taken during insufflation for laparoscopic surgery. The rib cage, lung, and diaphragm volumes are derived from the measurements of Cluzel et al. (2000), who measured them from MRI’s. The thoracoabdominal configuration at extreme and resting supine lung volumes are from Konno and Mead (1967).

Models were implemented using a program package written in the C language for the UNIX environment. Simulations were run on 64-bit Intel multiprocessor-based computers under the Linux operating system. The GNU Scientific Library was used to solve the differential equations of the biomechanical model, to find the roots of the implicit model equations, to do the abdominal volume integration, and for a spline approximation of the abdominal volume function.

For each condition of the linked neural network and biomechanical model, four trials were run with different random number seeds for the stochastic network model. A pairwise two-sided *t*-test with non-pooled SD was used for each variable, and the *p*-values were adjusted for multiple testing (Holm, 1979). A difference was considered significant if the adjusted *p*-value was less than 0.05.

## Results

The results are presented in two main parts. Section “Mechanical Model Implementation: Respiratory Muscles, Chest Wall, and Lungs” details the biomechanical model. Section “Brainstem Network Model Architecture and System Performance When Linked to the Biomechanical Model” describes the linkage between the biomechanical and neural network models and neuromechanical system behavior during various perturbations of the network.

### Mechanical model implementation: Respiratory muscles, chest wall, and lungs

The biomechanical model described below converts respiratory neural outputs in the form of spike trains representing lumbar, phrenic, expiratory laryngeal, and inspiratory laryngeal motor neuron activity generated by a stochastic model of the brainstem respiratory network deterministically into mechanical outputs such as lung volume, tracheal flow, and alveolar pressure for a supine male human. Lung volume is fed back to the network model to simulate pulmonary stretch receptors. The mechanical model components include (i) three-element Hill muscle models of the diaphragm and abdominal muscles (Hill, 1938), (ii) a model of the larynx based on the results of Tully et al. (1990, 1991) and Rohrer’s (1915) equation, and (iii) lung/diaphragm/ribcage/abdomen volume relationships modeled on the data of Grassino et al. (1978) and the analysis of Mead and Loring (1982).

The first two equations represent the entire mechanical model. Each term is a function of the motor outputs of the network model (*u*_di_, *u*_ab_, and *u*_lm_), and the diaphragm and abdominal wall volumes (*V*_di_ and *V*_ab_) and their time derivatives ($\dot{V}$_di_ and $\dot{V}$_ab_), and is defined by the subsequent equations. The parameters referenced in the model equations are listed in Table 1.

**Table 1:** **Parameters used in the biomechanical model**.

Parameter	Definition	Value	Units	Source
**FREE PARAMETERS USED IN THE MODEL**				
*C*_1_	Rib cage contribution to abdominal volume	0.369	Dimensionless	Derived from Grassino et al. (1978)
*C*_ab_	Compliance of the abdominal wall	0.108	L/cmH_2_O	Derived from Estenne et al. (1985)
*C*_L_	Compliance of the lung	0.201	L/cmH_2_O	Derived from Permutt and Martin (1960)
*c*_t_	Transverse chord of the abdominal wall	0.32	m	Derived from Song et al. (2006)
*D*	Diameter of the trachea	18	mm	Derived from Baier et al. (1977) and Kamel et al. (2009)
$f_{\text{a}}^{\text{TLC}}$	Obligatory ring fraction	0.15	Dimensionless	Mead and Loring (1982)
*F*_CEmax_	Maximal force capacity of the external oblique	33	N	Ratnovsky et al. (2003)
*F*_di_	Fraction of the diaphragm pressure expanding the rib cage via insertional forces	0.15	Dimensionless	Derived from Loring and Mead (1982)
*k*	Conversion factor from force to surface tension in abdominal muscle	0.68	m cmH_2_O/N	Derived from De Troyer et al. (1990) and Ratnovsky et al. (2003)
L_CEO_	Length of human transversus abdominis	19.1	cm	Gaumann et al. (1999)
$P_{\text{ica}}^{\text{abTLC}}$	Maximal expiratory pressure due to intercostal and accessory muscles at total lung capacity (TLC)	−135	cmH_2_O	Derived from Ratnovsky et al. (2008)
*R*_ab_	Passive resistance of the abdominal wall	1.5	cmH_2_O/(L/s)	Derived from Barnas et al. (1989)
*R*_di_	Passive resistance of the diaphragm	6	cmH_2_O/(L/s)	Derived from Barnas et al. (1989)
*R*_rc_	Passive resistance of the rib cage	2.7	cmH_2_O/(L/s)	Derived from Barnas et al. (1989)
*V*_c_	Mediastinal plus lung blood and tissue volume	1.756	L	Derived from Cluzel et al. (2000)
*V*_CEmax_	Maximal contractile velocity of the external oblique	34.7	cm/s	Ratnovsky et al. (2003)
$V_{\text{di}}^{\text{FRC}}$	Volume under diaphragm at functional residual capacity (FRC)	2.967	L	Derived from Cluzel et al. (2000)
${\dot{V}}_{\text{di}}^{\max}$	Maximal rate of change of volume under diaphragm	2.449	L/s	Derived from Goldman et al. (1978) and Chow and Darling (1999)
**FREE PARAMETERS USED IN CALCULATED PARAMETERS**				
$V_{\text{L}}^{\text{FRC}}$	Volume of the lung at FRC	2.29	L	Cluzel et al. (2000)
$V_{\text{rc}}^{\text{kmFRC}}$	Volume of the rib cage at FRC as a fraction of VC relative to residual volume (RV)	0.1282	Dimensionless	Konno and Mead (1967), Figure 14
$V_{\text{ab}}^{\text{kmFRC}}$	Volume of the abdominal wall at FRC as a fraction of VC relative to RV	0.0400	Dimensionless	Konno and Mead (1967), Figure 14
$V_{\text{ab}}^{\text{kmTLC}}$	Volume of the abdominal wall at TLC as a fraction of VC relative to RV	0.3391	Dimensionless	Konno and Mead (1967), Figure 14
VC	Vital capacity	5.370	L	Roca et al. (1998)
$\sigma_{\text{di}}^{\text{RV}}$	Passive recoil pressure of the diaphragm at RV	20	cmH_2_O	Derived from Agostoni et al. (1966), Grassino et al. (1978) and Siafakas et al. (1979)
$V_{\text{rc}}^{\text{FRC}}$	Volume of the rib cage at FRC	7.013	L	Derived from Cluzel et al. (2000)
$V_{\text{rc}}^{\text{kmTLC}}$	Volume of the rib cage at TLC as a fraction of VC relative to RV	0.6609	Dimensionless	Konno and Mead (1967), Figure 14
*f*_di_	Ratio of diaphragm length at TLC to RV	0.65	Dimensionless	Smith and Bellemare (1987)
*C*_rc_	Compliance of the rib cage	0.110	L/cmH_2_O	Derived from Gilroy et al. (1985)
**CALCULATED PARAMETERS USED IN CALCULATED PARAMETERS**				
$V_{\text{ab}}^{\text{RV}}$	Volume behind abdominal wall at RV		L	Eqs 32 and 33
$V_{\text{L}}^{\text{RV}}$	Volume of the lung at RV		L	Eq. 34
$\sigma_{\text{L}}^{\text{RV}}$	Passive recoil pressure of the lung at RV		cmH_2_O	Eq. 35
$\sigma_{\text{ab}}^{\text{RV}}$	Passive recoil pressure of the abdominal wall at RV		cmH_2_O	Eq. 36
$V_{\text{ab}}^{\text{FRC}}$	Volume behind abdominal wall at FRC		L	Eq. 37
$V_{\text{di}}^{\text{RV}}$	Volume under diaphragm at RV		L	Eq. 38
$V_{\text{ab}}^{\text{TLC}}$	Volume behind abdominal wall at TLC		L	Eq. 39
$V_{\text{L}}^{\text{TLC}}$	Volume of the lung at TLC		L	Eq. 40
$\sigma_{\text{L}}^{\text{TLC}}$	Passive recoil pressure of the lung at TLC		cmH_2_O	Eq. 41
$\sigma_{\text{ab}}^{\text{TLC}}$	Passive recoil pressure of the abdominal wall at TLC		cmH_2_O	Eq. 42
$\sigma_{\text{di}}^{\text{TLC}}$	Passive recoil pressure of the diaphragm at TLC		cmH_2_O	Eq. 43
$\sigma_{\text{rc}}^{\text{TLC}}$	Passive recoil pressure of the rib cage at TLC		cmH_2_O	Eq. 44
$\sigma_{\text{rc}}^{\text{RV}}$	Passive recoil pressure of the rib cage at RV		cmH_2_O	Eq. 45
$f_{\text{a}}^{\text{RV}}$	Fraction of the rib cage exposed to abdominal pressure at RV		Dimensionless	Eq. 46
**CALCULATED PARAMETERS USED IN THE MODEL**				
*V*_sum_	Sum of diaphragm, rib cage, and abdominal wall volume contributions		L	Eq. 47
*V*_ab0_	Volume behind abdominal wall at zero passive tension		L	Eqs 32 and 33
*V*_L0_	Volume of the lung at zero passive tension		L	Eq. 48
$K_{\text{di}}^{\text{psv}}$	Coefficient of passive diaphragm recoil pressure		cmH_2_O/L^2^	Eq. 49
$L_{\text{di}}^{\text{min}}$	Ratio of diaphragm length at zero volume to resting length		Dimensionless	Eq. 50
$P_{\text{ica}}^{\text{abRV}}$	Maximal expiratory pressure due to intercostal and accessory muscles at TLC		cmH_2_O	Eq. 51
$P_{\text{ica}}^{\text{diTLC}}$	Maximal inspiratory pressure due to intercostal and accessory muscles at TLC		cmH_2_O	Eq. 52
$\sigma_{\text{di}}^{\text{max}}$	Maximum active recoil pressure of the diaphragm		cmH_2_O	Eq. 53
$\sigma_{\text{rc}}^{\text{add}}$	Passive rib cage recoil pressure midway between volume limits		cmH_2_O	Eq. 54
$\sigma_{\text{rc}}^{\text{mul}}$	Rib cage sigmoid compliance coefficient		cmH_2_O	Eq. 55
*V*_di0_	Volume under diaphragm at zero tension		L	Eq. 56
$V_{\text{di}}^{\text{TLC}}$	Volume under diaphragm at TLC		L	Eq. 57
$V_{\text{rc}}^{\max}$	Upper limit of rib cage volume		L	Eq. 58
$V_{\text{rc}}^{\min}$	Lower limit of rib cage volume		L	Eq. 59
$V_{\text{rc}}^{\text{RV}}$	Volume of the rib cage at RV		L	Eq. 60
$V_{\text{rc}}^{\text{TLC}}$	Volume of the rib cage at TLC		L	Eq. 61
*V*_rc0_	Volume of the rib cage at zero tension		L	Eq. 62

#### Pressure balance on the rib cage

In Eq. 1, *P*_pl_ is the pleural pressure seen by the interior surface of the rib cage, *P*_ab−pl_ corrects for the fact that the rib cage sees abdominal pressure in the zone of apposition, *F*_di_σ_di_ is the equivalent pressure due to the insertional forces of the diaphragm on the lower ribs, *P*_ica_ is the equivalent pressure due to the intercostal and accessory muscles, and σ_rc_ is the recoil pressure of the rib cage that balances the sum of the other pressures.

(1) $$P_{\text{pl}}+P_{\text{ab}-\text{pl}}+F_{\text{di}}\sigma_{\text{di}}+P_{\text{ica}}=\sigma_{\text{rc}}$$

#### Pressure balance on the diaphragm

In Eq. 2, σ_ab_ is the abdominal pressure, the excess of which over the pleural pressure must be balanced by σ_di_, the recoil pressure of the diaphragm.

(2) $$\sigma_{\text{ab}}-P_{\text{pl}}=\sigma_{\text{di}}$$

#### Pleural pressure

In Eq. 3, tracheal flow ($\dot{V}$_L_) is the derivative of lung volume. *R*_rs_ is the resistance of the airway, which when multiplied by $\dot{V}$_L_ gives the pressure drop, by the hydraulic analog of Ohm’s law. σ_L_ is the recoil pressure of the lung.

(3) $$P_{\text{pl}}=-R_{\text{rs}}{\dot{V}}_{\text{L}}-\sigma_{\text{L}}$$

#### Lung volume

Lung volume is a function of *V*_di_ and *V*_ab_. Diaphragm volume, *V*_di_, is the volume above the level of the diaphragm insertions on the rib cage and below the dome of the diaphragm. Abdominal wall volume, *V*_ab_, is the volume between the abdominal wall and a frontal plane that coincides with the contracted position of the abdominal wall.

It has been commonly assumed that the abdominal contents are incompressible and that the abdominal cavity has only two movable walls – the diaphragm and the abdominal wall – and that therefore a displacement in one must be met by an equal and opposite displacement of the other (Grimby et al., 1976; Grassino et al., 1978; Macklem et al., 1978; Loring and Mead, 1982; Lichtenstein et al., 1992; Fitz-Clarke, 2007); in other words, that *V*_di_ + *V*_ab_ = *V*_sum_ (*V*_sum_ constant). Under this assumption, the abdominal volume completely determines the volume under the diaphragm, *V*_di_. Following Lichtenstein et al. (1992), *V*_di_ determines the static contractile pressure generated by the diaphragm at a given activation. However, experimental data (Grassino et al., 1978, Figure 4) show that both rib cage volume and abdominal volume affect this pressure. Therefore, in our model, we added a term, *C*_1_*V*_rc_, to the equation, allowing the rib cage and the abdominal wall to independently alter the volume under the diaphragm, effectively adding a third movable wall to the abdominal container, as discussed by Mead and Loring (1982).

The value of *C*_1_ was determined by fitting our model to published data (Grassino et al., 1978, Figure 4) giving diaphragm pressure as a function of rib cage and abdominal volume at a fixed diaphragm activation. Given a value of *C*_1_, our model equations will calculate the diaphragm pressure from the volumes. We then found the value of *C*_1_ that was the best fit of the calculated pressure values to the measured values. The resulting equation is *V*_di_ + *C*_1_*V*_rc_ + *V*_ab_ = *V*_sum_, which together with Eq. 20, gives us Eq. 4 for lung volume (*V*_L_).

(4) $$V_{\text{L}}=\frac{V_{\text{sum}}-\left(1+C_{1}\right)V_{\text{di}}-V_{\text{ab}}-C_{1}V_{\text{c}}}{C_{\text{1}}}$$

#### Airway resistance

Rohrer’s equation was used to calculate airway resistance (Rohrer, 1915; Hey and Price, 1982). The equation Pressure = *K*_1_ · Flow + *K*_2_ · Flow^2^ or, dividing through by flow, Resistance = *K*_1_ + *K*_2_ |Flow| was applied twice, once for laryngeal resistance (*k*_1_ + *k_2_*|$\dot{V}$_L_|) and once for the resistance of the oropharynx and lower airway (0.72 + 0.44|$\dot{V}$_L_|), to give Eq. 5. We used a value of *K*_2_ = 0.44 for the oropharynx and lower airway based on the assumption that *K*_2_ is 0 for the oropharynx (Renotte et al., 1998; Eq. 8), and 0.44 for the lower airway (Renotte et al., 1998, Table 2). Assuming *K*_1_ for the larynx is negligible during quiet breathing (see below), we used 0.34 for the lower airway and 0.38 for the oropharynx (Renotte et al., 1998, Table 2), for a total of 0.72.

The values of *k*_1_ and *k*_2_ for the larynx depend on the diameter of the glottis, as shown in Eqs 6–8. The parameter *D* is the diameter of the human trachea. The variable *u*_lm_ is the net laryngeal muscle activation, ranging from −1 (closed glottis) to 1 (open glottis). The variable *d* is the diameter of the glottis, or more precisely, the diameter of a circle with the same area as the opening of the glottis. The resting diameter (when *u*_lm_ = 0) is taken to be 10.9 mm (Baier et al., 1977; Brancatisano et al., 1983; D’Urzo et al., 1988), and is assumed (see Eq. 8) to change in proportion to *u*_lm_ (Tully et al., 1990, 1991).

The coefficient *k*_2_ given by Eq. 7 is calculated using the equation for flow through an orifice (Simpson, 1968, Table II). The value of *k*_2_ for the upper airway is different for inspiration and expiration (Renotte et al., 1998, Table 2), which we assume is due to changes in *d*. Assuming equal excursions from the resting diameter, we solved for the coefficient that would give us the reported values of *k*_2_, and got 0.167. This approach resulted in a resting value for *k*_2_ of 0.681.

We calculated the ratio of the mean resting value of *k*_1_ to the mean resting value of *k*_2_ (Tully et al., 1990, Table 2), and multiplied the ratio by 0.681 to get a resting value of *k*_1_ of 0.0035 for the larynx, which is small relative to *K*_1_ for the rest of the airway, justifying our assumption above that *K*_1_ for the larynx is negligible during quiet breathing.

The Darcy–Weisbach equation for pressure loss in a pipe and the Darcy friction factor for laminar flow tell us that the resistance is proportional to 1/*d*^4^ (Kreith et al., 2004), which we use in Eq. 6 for *k*_1_. Plugging in the resting value of *k*_1_ determined above and the resting value of *d*, we solved for the coefficient, which gives us 49.6.

$$\begin{array}{rcll} R_{\text{rs}} & =k_{1}+0.72+\left(k_{2}+0.44\right){\dot{V}}_{\text{L}} & & \text{(5)}\\ k_{1} & =\frac{49.6}{d^{4}} & & \text{(6)}\\ k_{2} & =0.167\left(\frac{D^{2}}{d^{4}}+\frac{d^{2}}{D^{2}}-1\right) & & \text{(7)}\\ d & =\left\{\begin{array}{cc} D,\; & u_{\text{lm}}>\frac{71}{109}\\ 10.9\left(1+u_{\text{lm}}\right),\; & -1\leq u_{\text{lm}}\leq \frac{71}{109}\\ 0,\; & u_{\text{lm}}<-1\\ \; \end{array}\right. & & \text{(8)} \end{array}$$

#### Abdominal pressure on the rib cage

A fraction *f*_a_ of the rib cage is exposed to abdominal pressure rather than pleural pressure. The recoil pressure of the diaphragm, σ_di_, is the difference between abdominal pressure and pleural pressure, so *P*_ab − pl_ adjusts the pressure seen by the rib cage for this difference.

(9) $$P_{\text{ab}-\text{pl}}=f_{\text{a}}\sigma_{\text{di}}$$

#### Abdominal fraction of the rib cage

At total lung capacity (TLC), none of the diaphragm is apposed to the rib cage (Mead and Loring, 1982), so we assumed that at all lung volumes, a portion of *V*_di_ equal to *V*_di_ at TLC $\left(V_{\text{di}}^{\text{TLC}}\right)$ does not contribute to the zone of apposition. The remainder $\left(V_{\text{di}}-V_{\text{di}}^{\text{TLC}}\right)$ is divided by the remainder plus the lung volume $\left(V_{\text{di}}-V_{\text{di}}^{\text{TLC}}-V_{\text{L}}\right)$ to give an estimate of the fraction of the rib cage surface above the diaphragm insertions that is exposed to abdominal pressure.

The “obligatory ring” below the diaphragm insertions, which is about 15% of the rib cage surface (Mead and Loring, 1982), is always exposed to abdominal pressure, and is represented by $f_{\text{a}}^{\text{TLC}}$ in Eq. 10. Our rib cage volume, *V*_rc_, is the volume above a plane through the diaphragm attachments (Cluzel et al., 2000, Figure 3). The estimate of 15% of the rib cage surface was in the context of a different definition of rib cage volume in which a change in rib cage volume is equal to the change in lung volume with the abdominal wall held still (Konno and Mead, 1967). This alternative definition implies a larger volume for the rib cage because it includes parts below the diaphragm insertions. From our volume equations, this means that rib cage volume is larger than ours by a factor of (1 + *C*_1_), so we divided the previously calculated fraction of the smaller rib cage by (1 + *C*_1_) to turn it into a fraction of the larger rib cage before adding it to $f_{\text{a}}^{\text{TLC}}.$

(10) $$f_{\text{a}}=\frac{1}{1+C_{1}}\left(\frac{V_{\text{di}}-V_{\text{di}}^{\text{TLC}}}{V_{\text{di}}-V_{\text{di}}^{\text{TLC}}+V_{\text{L}}}\right)+f_{\text{a}}^{\text{TLC}}$$

#### Recoil pressure of the lung

Equation 11 assumes a linear relationship between lung volume and recoil pressure. *C*_L_ is lung compliance. *V*_L0_ is the lung volume at zero recoil pressure, which we took to be equal to the residual volume (RV) after a maximal exhalation, the small recoil pressure remaining at RV being close enough to zero for our purposes (Permutt and Martin, 1960; Harris, 2005).

(11) $$\sigma_{\text{L}}=\frac{V_{\text{L}}-V_{\text{L0}}}{C_{\text{L}}}$$

#### Recoil pressure of the diaphragm

In Eq. 12, the term $u_{\text{di}}\sigma_{\text{di}}^{\text{max}}F_{\text{fl}}^{\text{di}}F_{\text{fv}}^{\text{di}}$ corresponds to the Hill muscle model (Ratnovsky et al., 2003, Eq. A6), except that $\sigma_{\text{di}}^{\text{max}}$ is a pressure rather than a force and $F_{\text{fl}}^{\text{di}}$ and $F_{\text{fv}}^{\text{di}}$ are functions of volume and its derivative (flow), respectively, rather than length and velocity.

We substituted pressure for force because, by Laplace’s (1808) Law, the pressure is proportional to the force when the curvature is constant, and the curvature of the human diaphragm dome does not change significantly with volume (Braun et al., (1982). Moreover, there is a constant ratio between diaphragm force and pressure in the dog (Kim et al., 1976).

Our substitution of volume for length (with an offset) is supported by the observation that the relationship between diaphragm pressure and length is not clearly different from linear when measured at RV, functional residual capacity (FRC), and TLC (Cluzel et al., 2000). To the extent that the action of the diaphragm resembles that of a piston (Kim et al., 1976), this linearity is expected. There is precedent for a Hill-style model in terms of pressure and flow for the respiratory system (Younes and Riddle, 1981).

In Eq. 12, *u*_di_ is phrenic activation of the diaphragm; $\sigma_{\text{di}}^{\text{max}}$ is static diaphragm recoil pressure at optimum length and maximum activation; *R*_di_$\dot{V}$_di_ is the pressure due to the passive resistance of the diaphragm.

(12) $$\sigma_{\text{di}}=u_{\text{di}}\sigma_{\text{di}}^{\text{max}}F_{\text{fl}}^{\text{di}}F_{\text{fv}}^{\text{di}}+\sigma_{\text{di}}^{\text{psv}}+R_{\text{di}}{\dot{V}}_{\text{di}}$$

#### Passive recoil pressure of the diaphragm

In Eq. 13, $\sigma_{\text{di}}^{\text{psv}}$ is the passive transdiaphragmatic pressure as a function of diaphragm volume. This pressure is taken to be zero at resting diaphragm volume $V_{\text{di}}^{\text{FRC}}$ (Agostoni and Rahn, 1960) and below, and quadratic above (Reid et al., 1987).

(13) $$\sigma_{\text{di}}^{\text{psv}}=\left\{\begin{array}{cc} K_{\text{di}}^{\text{psv}}.{\left(V_{\text{di}}-V_{\text{di}}^{\text{FRC}}\right)}^{2},\; & V_{\text{di}}>V_{\text{di}}^{\text{FRC}}\\ 0,\; & V_{\text{di}}\leq V_{\text{di}}^{\text{FRC}}\\ \; \end{array}\right.$$

#### Volume-pressure relationship of the diaphragm

In Eq. 14, $F_{\text{fl}}^{\text{di}}$ is the static pressure-volume relationship of the diaphragm (corresponding to Ratnovsky et al., 2003, Eq. A7 with the relative length replaced by a linear function of volume as described above). The parameter *V*_di0_ is the volume under the diaphragm at the resting length. This is taken to be equal to the diaphragm volume at RV, based on the observation that the “highest Pdi twitch amplitude was recorded at RV” (Smith and Bellemare, 1987). The parameter $L_{\text{di}}^{\min}$ is the length of the diaphragm at zero volume (i.e., when the diaphragm is flat) divided by the resting length, and is calculated by assuming that the length of the diaphragm at TLC is 65% of that at RV (Smith and Bellemare, 1987).

(14) $$F_{\text{fl}}^{\text{di}}=\exp\left(-0.5{\left(\frac{\frac{1-L_{\text{di}}^{\text{min}}}{V_{\text{di0}}}V_{\text{di}}+L_{\text{di}}^{\text{min}}-1.05}{0.19}\right)}^{2}\right)$$

#### Pressure-flow relationship of the diaphragm

In Eq. 15, $F_{\text{fv}}^{\text{di}}$ is the pressure-flow relationship of the diaphragm, with the velocity replaced by flow as discussed above (Hatze, 1981; Rosen et al., 1999; Artemiadis and Kyriakopoulos, 2005). The variable $\dot{V}$_di_ is the time derivative of the volume under the diaphragm. The maximum rate of change of diaphragm volume, ${\dot{V}}_{\text{di}}^{\text{max}},$ was derived from data which gives transdiaphragmatic pressure as a function of flow at several levels of diaphragm activation up to 45% (Goldman et al., 1978, Figure 6). Because the rib cage was held still, the flow represents the rate of change of diaphragm volume. Fitting the curves to a Hill-style relation between pressure and flow (Younes and Riddle, 1981), there is a maximum flow (where the pressure goes to zero) for each level of diaphragm activation. Experimental results suggest that the maximum flow increases somewhat linearly to 80% activation and then levels off (Chow and Darling, 1999). We used that assumption together with the results for the maximum flow at lower activations to compute a maximum flow at 100% activation, which we use for ${\dot{V}}_{\text{di}}^{\text{max}}.$

(15) $$F_{\text{fv}}^{\text{di}}=\frac{0.1433}{0.1074+\exp\left(-1.409\;\text{sinh}\left(3.2\frac{{\dot{V}}_{\text{di}}}{{\dot{V}}_{\text{di}}^{\max}}+1.6\right)\right)}$$

#### Pressure of the intercostal and accessory muscles

In Eq. 16, *P*_ica_ is the effective pressure generated by the intercostal and accessory muscles; positive values act to expand the rib cage.

In Eq. 17, $P_{\text{ica}}^{\text{di}}$ is the pressure due to the action of the inspiratory intercostals, which are assumed to be inactive when the diaphragm volume is above $V_{\text{di}}^{\text{FRC}}$ (low lung volumes). Below $V_{\text{di}}^{\text{FRC}},$ the pressure exerted by the inspiratory intercostals is assumed to be proportional to the activation of the diaphragm (*u*_di_), and the proportionality constant itself is assumed to scale linearly from 0 at $V_{\text{di}}^{\text{FRC}}$ to its maximum value of $P_{\text{ica}}^{\text{diTLC}}$ at $V_{\text{di}}^{\text{TLC}}.$ The parameter $P_{\text{ica}}^{\text{diTLC}}$ was calculated as the pressure necessary to complete the pressure balance on the rib cage at TLC.

In Eq. 18, $P_{\text{ica}}^{\text{ab}}$ is the pressure due to the action of the expiratory intercostals, which is assumed to be proportional to the abdominal muscle activation (*u*_ab_); the proportionality constant itself is assumed to scale linearly with the rib cage volume, changing from $P_{\text{ica}}^{\text{abRV}}$ at RV to $P_{\text{ica}}^{\text{abTLC}}$ at TLC. In Eq. 18, $P_{\text{ica}}^{\text{abTLC}}$ is the pressure due to the expiratory intercostals at TLC and maximal abdominal activation. This parameter’s value was calculated by taking the mean male maximal mouth pressure at TLC (from Ratnovsky et al., 2008, Table 1) and subtracting it from the rib cage recoil pressure at TLC. $P_{\text{ica}}^{\text{abRV}}$ is the pressure due to the expiratory intercostals at RV and maximal abdominal activation. We calculated this parameter by solving the model equations for *P*_ica_ while assuming RV conditions. This gives us the intercostal pressure necessary to reach RV.

$$\begin{array}{rcll} P_{\text{ica}} & =P_{\text{ica}}^{\text{di}}+P_{\text{ica}}^{\text{ab}} & & \text{(16)}\\ P_{\text{ica}}^{\text{di}} & =\left\{\begin{array}{cc} u_{\text{di}}P_{\text{ica}}^{\text{diTLC}}\frac{V_{\text{di}}-V_{\text{di}}^{\text{FRC}}}{V_{\text{di}}^{\text{TLC}}-V_{\text{di}}^{\text{FRC}}},\; & V_{\text{di}}<V_{\text{di}}^{\text{FRC}}\\ \text{0,}\; & V_{\text{di}}\geq V_{\text{di}}^{\text{FRC}}\\ \; \end{array}\right. & & \text{(17)}\\ P_{\text{ica}}^{\text{ab}} & =u_{\text{ab}}\left(P_{\text{ica}}^{\text{abRV}}+\frac{V_{\text{rc}}-V_{\text{rc}}^{\text{RV}}}{V_{\text{rc}}^{\text{TLC}}-V_{\text{rc}}^{\text{RV}}}\left(P_{\text{ica}}^{\text{abTLC}}-P_{\text{ica}}^{\text{abRV}}\right)\right) & & \text{(18)} \end{array}$$

#### Recoil pressure of the rib cage

The volume of the rib cage is assumed to be a sigmoid function of the recoil pressure of the rib cage, σ_rc_. With increasing pressure the volume asymptotically approaches a maximum $\left(V_{\text{rc}}^{\text{max}}\right),$ and with decreasing pressure it asymptotically approaches a minimum $\left(V_{\text{rc}}^{\text{min}}\right).$ A generalized logistic function is used for the sigmoid, giving *V*_rc_ as a function of σ_rc_; that equation is solved for σ_rc_ to give the first part of Eq. 19. The final term of Eq. 19 is the pressure due to the passive resistance of the rib cage (*R*_rc_) and the rate of change of its volume ($\dot{V}$_rc_) The parameter $\sigma_{\text{rc}}^{\text{mul}}$ controls the maximum slope of the sigmoid; the slope is the compliance of the rib cage. It is calculated to make the compliance equal to *C*_rc_/(1 + *C*_1_). The factor of (1 + *C*_1_) appears because *C*_rc_ is for a rib cage volume defined differently than *V*_rc_. *C*_rc_ is the compliance of the rib cage, an average of values for three sitting subjects (Gilroy et al., 1985, Table 1).

(19) $$\sigma_{\text{rc}}=\sigma_{\text{rc}}^{\text{mul}}\log\left(\frac{V_{{}_{\text{rc}}}^{\max}-V_{\text{rc}}}{V_{\text{rc}}-V_{\text{rc}}^{\min}}\right)+\sigma_{\text{rc}}^{\text{add}}+R_{\text{rc}}{\dot{V}}_{\text{rc}}$$

#### Volume of the rib cage

The rib cage volume (*V*_rc_) is the sum of the lung volume (*V*_L_), the volume under the diaphragm (*V*_di_), and the volume of the mediastinum and the lung blood and tissue (*V*_c_).

(20) $$V_{\text{rc}}=V_{\text{L}}+V_{\text{di}}+V_{\text{c}}$$

#### Recoil pressure of the abdominal wall

The abdominal wall is modeled as a surface with a circular segment cross-section in each transverse plane, all with the same radius, and a circular segment cross-section in each sagittal plane, all with another radius. The volume behind the abdominal wall, *V*_ab_, is bounded by this surface and by a frontal plane. The values for the sagittal (*r*_s_) and transverse (*r*_t_) radii were derived from measurements taken during insufflation for laparoscopic surgery in humans (see Figure 3, Song et al., 2006). We fit exponential curves to the data points and the resulting relationship between the fitted sagittal and transverse radii was found to be well approximated by a linear function: *r*_s_ = 8.00 *r*_t_ − 1.10. The length of the longest transverse chord in the bounding frontal plane (*c*_t_) was found which gave the volume change stated in the paper.

In Eq. 21, $u_{\text{ab}}F_{\text{CEmax}}F_{\text{fl}}^{\text{ab}}F_{\text{fv}}^{\text{ab}}$ is the Hill muscle model equation (Ratnovsky et al., 2003, Eq. A6); *u*_ab_ is the activation of the diaphragm by the lumbar motor neurons; *F*_CEmax_ is the maximal force capacity of the contractile element for a 1.5 cm^2^ cross-section of canine external oblique muscle (Ratnovsky et al., 2003, Table 1). The constant *k* converts from a force to a surface tension, and (1/*r*_s_ + 1/*r*_t_) converts the surface tension to a pressure, using the Law of Laplace (Laplace, 1808). The second term on the right, (*V*_ab_ − *V*_ab0_)/*C*_ab_, is the passive recoil pressure of the abdominal wall. *V*_ab0_ is the volume behind the abdominal wall at which the recoil pressure is 0. This was taken to be equal to $V_{\text{ab}}^{\text{FRC}},$ since we assume a supine position. *C*_ab_ is the compliance of the abdominal wall. The final term is the pressure due to the passive resistance of the abdominal wall (*R*_ab_) and the rate of change of abdominal volume ($\dot{V}$_ab_).

(21) $$\sigma_{\text{ab}}=u_{\text{ab}}F_{\text{CEmax}}F_{\text{fl}}^{\text{ab}}F_{\text{fv}}^{\text{ab}}.\left(\frac{k}{r_{\text{t}}}+\frac{k}{r_{\text{s}}}\right)+\frac{V_{\text{ab}}-V_{\text{ab0}}}{C_{\text{ab}}}+R_{\text{ab}}{\dot{V}}_{\text{ab}}$$

In Eq. 22, $F_{\text{fl}}^{\text{ab}}$ is the static force-length relationship of the abdominal wall (Ratnovsky et al., 2003, Eq. A7); *L*_CE_ is the length of the transversus abdominis; *L*_CE0_ is its resting length.

(22) $$F_{\text{fl}}^{\text{ab}}=\exp\left(-0.5{\left(\frac{\frac{L_{\text{CE}}}{L_{\text{CE0}}}-1.05}{0.19}\right)}^{2}\right)$$

In Eq. 23, $F_{\text{fv}}^{\text{ab}}$ is the force-velocity relationship of the abdominal wall muscles (Hatze, 1981; Rosen et al., 1999; Artemiadis and Kyriakopoulos, 2005). The variable ${\dot{L}}_{\text{CE}}$ is the velocity of the contractile element (the time derivative of *L*_CE_) and the parameter *V*_CEmax_ is the maximal contractile velocity of canine external oblique muscle (Ratnovsky et al., 2003, Table 1).

(23) $$F_{\text{fv}}^{\text{ab}}=\frac{0.1433}{0.1074+\exp\left(-1.409\sinh\left(3.2\frac{{\dot{L}}_{\text{CE}}}{V_{\text{CEmax}}}+1.6\right)\right)}$$

#### Length-volume relationship of the abdominal wall

Equations 24 through 31 calculate the length of the abdominal muscle (*L*_CE_) from the volume behind the abdominal wall (*V*_ab_). Eqs 26 through 31 calculate *V*_ab_ as a function of *r*_t_ (the transverse radius); Eq. 25 uses the inverse of the resulting function to calculate *r*_t_ from *V*_ab_; Eq. 24 calculates *L*_CE_ from *r*_t_. In practice, the function *V*_ab_(*r*_t_) is pre-calculated, and approximated and inverted with a spline, and the spline is used to evaluate *r*_t_(*V*_ab_) during simulation.

$$\begin{array}{rcll} L_{\text{CE}} & =100r_{\text{t}}\sin^{-1}\left(\frac{c_{\text{t}}}{2r_{\text{t}}}\right) & & \text{(24)}\\ r_{\text{t}}\left(V_{\text{ab}}\right) & =\left\{\begin{array}{cc} V^{-1}\left(V_{\text{ab}}\right),\; & V_{\text{ab}}>0\\ 0.5c_{\text{t}},\; & V_{\text{ab}}\leq 0\\ \; \end{array}\right. & & \text{(25)}\\ V\left(r_{\text{t}}\right) & =\overset{c_{\text{s}}∕2}{\underset{-c_{\text{s}}∕2}{\int }}A\left(r_{\text{t}},y\right)dy & & \text{(26)} \end{array}$$

In Eq. 27, the Pythagorean Theorem is applied in the midsagittal plane to get *c*_s_, the length of the chord that connects the ends of the abdominal wall in the frontal plane that serves as a boundary of the abdominal wall volume.

(27) $$c_{\text{s}}=2\sqrt{{r_{\text{s}}}^{2}-{\left(h_{0}-r_{\text{s}}\right)}^{2}}$$

In Eq. 28, the Pythagorean Theorem is applied in a transverse plane to get *h*_0_, the distance from the peak of the abdominal wall to the frontal plane that serves as a boundary of the abdominal wall volume.

(28) $$h_{\text{0}}=r_{\text{t}}-\sqrt{r_{\text{t}}^{2}-{\left(\frac{c_{\text{t}}}{2}\right)}^{2}}$$

In Eq. 29, the formula for the area of a circular segment is applied in the transverse plane at a distance *y* from the peak of the abdominal wall to get the area between the abdominal wall and the boundary frontal plane.

(29) $$A=\frac{r_{\text{t}}^{2}}{2}\left(2\cos^{-1}\left(1-\frac{h}{r_{\text{t}}}\right)+\sin\left(2\cos^{-1}\left(\frac{h}{r_{\text{t}}}-1\right)\right)\right)$$

In Eq. 30, the Pythagorean theorem is applied in the midsagittal plane to get *h*, the distance from the abdominal wall to the boundary frontal plane at a distance *y* in the craniocaudal direction from the peak of the abdominal wall.

(30) $$h=\sqrt{r_{\text{s}}^{2}-y^{2}}-\sqrt{r_{s}^{2}-{\left(\frac{c_{\text{s}}}{2}\right)}^{2}}$$

Equation 31 is the relation between the sagittal radius (*r*_s_) and the transverse radius (*r*_t_) derived from the results in Song et al. (2006).

(31) $$r_{\text{s}}=8.00479r_{\text{t}}-1.10158$$

#### Equations for calculated parameters

$$\begin{array}{rcll} & \sigma_{\text{ab}}\left(u_{\text{ab}}=1,\;V_{\text{ab}}^{\text{RV}},\;{\dot{V}}_{\text{ab}}=0,\;V_{\text{ab0}}\right)=\sigma_{\text{ab}}^{\text{RV}} & & \text{(32)} \end{array}$$

$$\begin{array}{rcll} & V_{\text{ab0}}=V_{\text{ab}}^{\text{RV}}+V_{\text{ab}}^{\text{kmFRC}}\cdot \text{VC} & & \text{(33)} \end{array}$$

$$\begin{array}{rcll} & V_{\text{L}}^{\text{RV}}=V_{\text{L}}^{\text{FRC}}-\left(V_{\text{rc}}^{\text{kmFRC}}+V_{\text{ab}}^{\text{kmFRC}}\right)\cdot \text{VC} & & \text{(34)} \end{array}$$

$$\begin{array}{rcll} & \sigma_{\text{L}}^{\text{RV}}=\frac{V_{\text{L}}^{\text{RV}}-V_{\text{L0}}}{C_{\text{L}}} & & \text{(35)} \end{array}$$

$$\begin{array}{rcll} & \sigma_{\text{ab}}^{\text{RV}}=\sigma_{\text{di}}^{\text{RV}}-\sigma_{\text{L}}^{\text{RV}} & & \text{(36)} \end{array}$$

$$\begin{array}{rcll} & V_{\text{ab}}^{\text{FRC}}=V_{\text{ab}}^{\text{RV}}+V_{\text{ab}}^{\text{kmFRC}}\cdot \text{VC} & & \text{(37)} \end{array}$$

$$\begin{array}{rcll} & V_{\text{sum}}=V_{\text{di}}^{\text{FRC}}+C_{\text{1}}V_{\text{rc}}^{\text{FRC}}+V_{\text{ab}}^{\text{FRC}} & & \text{(38)} \end{array}$$

$$\begin{array}{rcll} & V_{\text{di}}^{\text{RV}}=V_{\text{sum}}-V_{\text{ab}}^{\text{RV}}-C_{\text{1}}V_{\text{rc}}^{\text{RV}} & & \text{(39)} \end{array}$$

$$\begin{array}{rcll} & V_{\text{ab}}^{\text{TLC}}=V_{\text{ab}}^{\text{FRC}}+\left(V_{\text{ab}}^{\text{kmTLC}}-V_{\text{ab}}^{\text{kmFRC}}\right)\cdot \text{VC} & & \text{(40)} \end{array}$$

$$\begin{array}{rcll} & V_{\text{L}}^{\text{TLC}}=V_{\text{rc}}^{\text{TLC}}-V_{\text{di}}^{\text{TLC}}-V_{\text{c}} & & \text{(41)} \end{array}$$

$$\begin{array}{rcll} & \sigma_{\text{L}}^{\text{TLC}}=\frac{V_{\text{L}}^{\text{TLC}}-V_{\text{L0}}}{C_{\text{L}}} & & \text{(42)} \end{array}$$

$$\begin{array}{rcll} & \sigma_{\text{ab}}^{\text{TLC}}=\frac{V_{\text{ab}}^{\text{TLC}}-V_{\text{ab0}}}{C_{\text{ab}}} & & \text{(43)} \end{array}$$

$$\begin{array}{rcll} & \sigma_{\text{di}}^{\text{TLC}}=\sigma_{\text{L}}^{\text{TLC}}-\sigma_{\text{ab}}^{\text{TLC}} & & \text{(44)} \end{array}$$

$$\begin{array}{rcll} & \sigma_{\text{rc}}^{\text{TLC}}=\sigma_{\text{rc}}^{\text{mul}}\log\left(\frac{V_{\text{rc}}^{\max}-V_{\text{rc}}^{\text{TLC}}}{V_{\text{rc}}^{\text{TLC}}-V_{\text{rc}}^{\text{min}}}\right)+\sigma_{\text{rc}}^{\text{add}} & & \text{(45)} \end{array}$$

$$\begin{array}{rcll} & \sigma_{\text{rc}}^{\text{RV}}=\sigma_{\text{rc}}^{\text{mul}}\log\left(\frac{V_{\text{rc}}^{\text{max}}-V_{\text{rc}}^{\text{RV}}}{V_{\text{rc}}^{\text{RV}}-V_{\text{rc}}^{\text{min}}}\right)+\sigma_{\text{rc}}^{\text{add}} & & \text{(46)} \end{array}$$

$$\begin{array}{rcll} & f_{\text{a}}^{\text{RV}}=\frac{1}{1+C_{\text{1}}}\left(\frac{V_{\text{di}}^{\text{RV}}-V_{\text{di}}^{\text{TLC}}}{V_{\text{di}}^{\text{RV}}-V_{\text{di}}^{\text{TLC}}+V_{\text{L}}^{\text{RV}}}\right)+f_{\text{a}}^{\text{TLC}} & & \text{(47)} \end{array}$$

$$\begin{array}{rcll} & V_{\text{L0}}=V_{\text{L}}^{\text{RV}} & & \text{(48)} \end{array}$$

$$\begin{array}{rcll} & K_{\text{di}}^{\text{psv}}=\frac{\sigma_{\text{di}}^{\text{RV}}}{{\left(V_{\text{di}}^{\text{RV}}-V_{\text{di}}^{\text{FRC}}\right)}^{2}} & & \text{(49)} \end{array}$$

$$\begin{array}{rcll} & L_{\text{di}}^{\text{min}}=\frac{V_{\text{di}}^{\text{TLC}}-f_{\text{di}}V_{\text{di}}^{\text{RV}}}{V_{\text{di}}^{\text{TLC}}-V_{\text{di}}^{\text{RV}}∕1.05} & & \text{(50)} \end{array}$$

$$\begin{array}{rcll} & P_{\text{ica}}^{\text{abRV}}=\sigma_{\text{L}}^{\text{RV}}+\sigma_{\text{rc}}^{\text{RV}}-\left(f_{\text{a}}^{\text{RV}}+F_{\text{di}}\right)\sigma_{\text{di}}^{\text{RV}} & & \text{(51)} \end{array}$$

$$\begin{array}{rcll} & P_{\text{ica}}^{\text{diTLC}}=\sigma_{\text{L}}^{\text{TLC}}+\sigma_{\text{rc}}^{\text{TLC}}-\left(f_{\text{a}}^{\text{TLC}}+F_{\text{di}}\right)\sigma_{\text{di}}^{\text{TLC}} & & \text{(52)} \end{array}$$

$$\begin{array}{rcll} & \sigma_{\text{di}}\left(u_{\text{di}}=1,\;V_{\text{di}}^{\text{TLC}},\;{\dot{V}}_{\text{di}}=0,\;\sigma_{\text{di}}^{\text{max}}\right)=\sigma_{\text{di}}^{\text{TLC}} & & \text{(53)} \end{array}$$

$$\begin{array}{rcll} & \sigma_{\text{rc}}^{\text{add}}=\sigma_{\text{rc}}^{\text{mul}}\log\left(\frac{V_{\text{rc0}}-V_{\text{rc}}^{\text{min}}}{V_{\text{rc}}^{\text{max}}-V_{\text{rc0}}}\right) & & \text{(54)} \end{array}$$

$$\begin{array}{rcll} & \sigma_{\text{rc}}^{\text{mul}}=\frac{\left(V_{\text{rc}}^{\text{max}}-V_{\text{rc}}^{\text{min}}\right)\left(1+C_{\text{1}}\right)}{4C_{\text{rc}}} & & \text{(55)} \end{array}$$

$$\begin{array}{rcll} & V_{\text{di0}}=V_{\text{di}}^{\text{RV}} & & \text{(56)} \end{array}$$

$$\begin{array}{rcll} & V_{\text{di}}^{\text{TLC}}=V_{\text{sum}}-V_{\text{ab}}^{\text{TLC}}-C_{\text{1}}V_{\text{rc}}^{\text{TLC}} & & \text{(57)} \end{array}$$

$$\begin{array}{rcll} & V_{\text{rc}}^{\text{max}}=V_{\text{rc}}^{\text{TLC}}+0.05\left(V_{\text{rc}}^{\text{TLC}}-V_{\text{rc}}^{\text{RV}}\right) & & \text{(58)} \end{array}$$

$$\begin{array}{rcll} & V_{\text{rc}}^{\text{min}}=V_{\text{rc}}^{\text{RV}}-0.99\left(V_{\text{rc}}^{\text{TLC}}-V_{\text{rc}}^{\text{RV}}\right) & & \text{(59)} \end{array}$$

$$\begin{array}{rcll} & V_{\text{rc}}^{\text{RV}}=V_{\text{rc}}^{\text{FRC}}-\frac{V_{\text{rc}}^{\text{kmFRC}}\cdot \text{VC}}{1+C_{\text{1}}} & & \text{(60)} \end{array}$$

$$\begin{array}{rcll} & V_{\text{rc}}^{\text{TLC}}=V_{\text{rc}}^{\text{FRC}}+\frac{\left(V_{\text{rc}}^{\text{kmTLC}}-{\text{V}}_{\text{rc}}^{\text{kmFRC}}\right)\cdot \text{VC}}{1+C_{\text{1}}} & & \text{(61)} \end{array}$$

$$\begin{array}{rcll} & V_{\text{rc0}}=V_{\text{rc}}^{\text{FRC}} & & \text{(62)} \end{array}$$

### Brainstem network model architecture and system performance when linked to the biomechanical model

**Figure 1:**
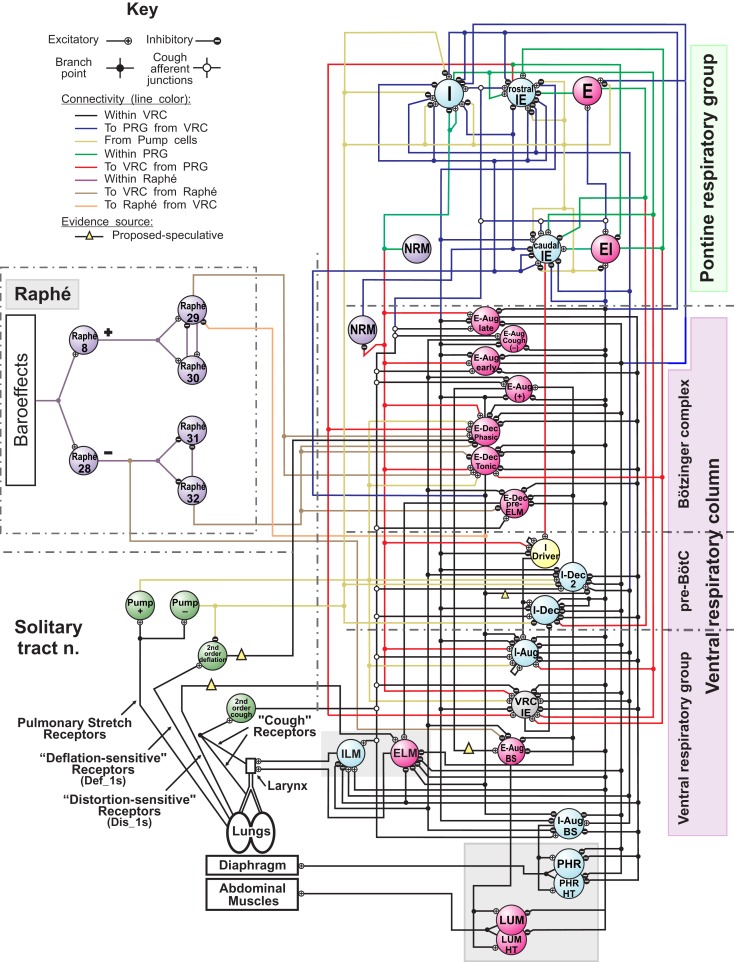
**Schematic of the raphé-pontomedullary respiratory network model used in this study**. The model extends that used in Poliaček et al. (2011) and Rybak et al. (2008) and follows labeling conventions enumerated therein. Parameters for the represented cell populations (large colored circles) and connections (see Key) are listed in Tables 2 and 4. Parameters for the I-Driver population with conditional bursting pacemaker properties were as described previously (Rybak et al., 2008; Poliaček et al., 2011). Abbreviations of brainstem regions or “compartments”: pre-BötC, pre-Bötzinger complex; VRC or VRG, ventral respiratory column or group; PRG, pontine respiratory group. Abbreviations of most populations were as enumerated in Table 1 of Rybak et al. (2008): Aug and Dec: neurons with augmenting or decrementing activity patterns, respectively, during the indicated phase (I-inspiratory; E-expiratory) of maximum firing rate. BS, bulbo-spinal; ELM, expiratory laryngeal motoneurons; EI, neurons with a peak firing rate during the E-to-I phase transition; IE, neurons with a peak firing rate during the I-to-E phase transition; ILM, inspiratory laryngeal motoneurons; NRM, non-respiratory-modulated neurons. Two phrenic motor neuron populations with different threshold ranges innervated the diaphragm (PHR, PHR-HT: high threshold); two lumbar motor neuron populations activated the abdominal muscle (LUM, LUM-HT: high threshold). I-Dec_2, second inhibitory I population in the VRC (e.g., see Ott et al., 2012); Lung Def_1s, Lung Dis_1s, Deflation 2nd order: lung deflation-sensing neuronal circuit elements. See text for further discussion.

The computational model of the pontomedullary respiratory network (Figure 1) instantiated the hypothesis (Shannon et al., 1998; Rybak et al., 2008) that airway cough receptors affect several neuron populations in the ventral respiratory column (VRC) and pontine respiratory group (PRG) via cough 2nd order neurons. Evoked changes reconfigured the respiratory network to produce the cough motor pattern through the same VRC neurons involved in providing drive to respiratory muscles during normal breathing. The model incorporated recent enhancements (Poliaček et al., 2011) and additional neuron populations and other changes as detailed in Tables 2–4.

**Table 2:** **Population parameters for network model with adjusted (Δ) and additional (+) neuronal populations modified from Poliaček et al. (2011)**.

Population name	Size	Resting threshold (mV)	THO variability (mV)	Membrane time constant	Post-spike increase in G_K+_	Post-spike *G*_K+_ time constant (ms)	Adaptation threshold increase	Adaptation (ms)	Noise amplitude	DC (mV)
	*N*	*THO*		*TMEM*	*B*	*TGK*	*C*	*TTH*		
I-pons	100	10.0	1.0	9.0	20.0	7.0	0.0	500.0	0.03	2.0
rostral IE-pons	100	10.0	1.0	9.0	20.0	7.0	0.0	500.0	0.3	5.0
caudal IE-pons	100	10.0	1.0	9.0	20.0	7.0	0.0	500.0	0.3	5.0
E-pons	100	10.0	1.0	9.0	20.0	7.0	0.0	500.0	0.3	13.0
EI-pons	100	10.0	1.0	9.0	20.0	7.0	0.0	500.0	0.3	20.0
NRM-pons	100	10.0	1.0	9.0	20.0	7.0	0.0	500.0	0.03	25.0
NRM-BötC	300	10.0	1.0	9.0	20.0	7.0	0.0	500.0	0.03	25.0
ΔI-DRIVER	300	See Table A2 in Rybak et al. (2008) for I-Driver neuron properties								
ΔI-Dec	300	10.0	1.0	6.0	75.0	8.5	0.9	1500.0	0.2	20.0
+I-Dec_2	300	10.0	1.0	6.0	75.0	8.5	0.8	1200.0	0.2	5.0
ΔI-Aug	300	12.0	3.0	6.0	75.0	5.0	0.0	5000.0	0.6	18.0
ΔVRC IE	99	18.0	2.0	9.0	50.0	5.0	0.0	1000.0	0.075	0.0
ΔE-Dec-Phasic	300	9.0	1.0	6.0	75.0	8.5	0.9	1500.0	0.1	4.0
ΔE-Dec-Tonic	300	8.0	1.0	9.0	50.0	3.8	0.8	2000.0	0.3	0.0
E-Aug-early	300	10.0	1.0	6.0	27.0	2.5	0.0	500.0	0.3	30.0
E-Aug-late	300	10.0	1.0	9.0	27.0	2.5	0.0	500.0	0.1	27.0
ΔE-Aug-Cough	300	12.0	2.0	9.0	75.0	7.5	0.0	500.0	0.2	0.0
+E-Aug (+)	300	10.0	2.0	6.0	75.0	7.5	0.1	1000.0	0.5	20.0
ΔE-Aug-BS (+)	300	12.0	3.0	6.0	100.0	6.0	0.1	1000.0	0.5	0.0
ΔPump (+)	300	5.0	0.5	6.0	25.0	3.8	0.08	500.0	0.1	0.0
ΔPump (-)	300	5.0	0.0	6.0	25.0	3.8	0.08	500.0	0.1	0.0
ΔI-Aug-BS	300	12.0	3.0	6.0	75.0	5.0	0.0	5000.0	0.5	0.0
+Phrenic	210	10.0	2.0	5.0	200.0	6.0	0.08	500.0	0.5	0.0
+Phrenic-HT	70	16.0	2.0	60.0	200.0	5.0	0.08	500.0	0.5	0.0
ΔLumbar	210	15.0	2.0	6.0	75.0	7.5	0.08	500.0	0.5	0.0
+Lumbar-HT	70	18.0	2.0	30.0	200.0	7.5	0.1	500.0	0.5	0.0
ΔILM	300	20.0	1.0	6.0	25.0	3.8	0.08	500.0	0.1	2.0
ΔE-Dec-pre-ELM	300	11.0	0.0	6.0	100.0	6.0	0.8	500.0	0.5	1.0
ΔELM	300	18.0	2.0	6.0	100.0	6.0	0.9	100.0	0.5	0.0
ΔLUNG PSRs	300	11.0	1.0	9.0	20.0	7.0	0.0	500.0	0.5	0.0
+Lung deflation receptors (Def_1)	300	10.0	1.0	9.0	20.0	7.0	0.0	500.0	0.5	0.0
+Lung distortion receptors (Dis_1)	300	10.0	1.0	9.0	20.0	7.0	0.0	500.0	0.5	0.0
>Cough 2nd order	100	10.0	1.0	9.0	20.0	7.0	0.3	500.0	0.1	0.0
ΔDeflation 2nd order	300	8.0	1.0	9.0	27.0	2.5	0.5	1000.0	0.3	0.0
Raphé 8	100	10.0	1.0	9.0	20.0	7.0	0.0	500.0	0.01	0.0
Raphé 28	100	10.0	1.0	9.0	20.0	7.0	0.0	500.0	0.1	0.0
Raphé 29	100	10.0	1.0	9.0	20.0	7.0	0.0	500.0	0.5	0.0
Raphé 30	100	10.0	1.0	9.0	20.0	7.0	0.0	500.0	0.5	0.0
Raphé 31	100	10.0	1.0	9.0	20.0	7.0	0.0	500.0	0.1	10.0
Raphé 32	100	10.0	1.0	9.0	20.0	7.0	0.0	500.0	0.1	10.0

*Variable names used by MacGregor (1987) are in italics. All values representing voltages are relative to the resting potential, which is considered equal to zero. *N* is the number of neurons simulated in each population. *THO*, the resting threshold, is normally distributed in the population around the value of THO with a standard deviation equal to the “THO variability” value. *TMEM* is the membrane time constant. *B* is the amplitude of the post-spike increase in potassium conductance. *TGK* is the time constant of the potassium conductance decay following an action potential. *C* and *TTH* define the change in threshold associated with spike adaptation. *C* is the ratio of the threshold increase to the membrane potential increase; its value is between 0 and 1. *TTH* is the time constant of the rise in threshold with spike adaptation. *Noise Amplitude*: each cell has an internal noise generator that acts like two synapses, one with an equilibrium potential of 70 mV above resting and the other with −70 mV. Each acts like it has an incoming firing probability of 0.05 per time step, and a synapse time constant of 1.5 ms. This parameter is the conductance that gets added to the synapse conductance on each (virtual) spike. *DC*: an injected current will raise the membrane potential by an amount that is inversely proportional to the membrane conductance. Instead of being specified directly as a current, this parameter is specified in mV, and it is interpreted as the current that is required to raise the membrane potential by the specified number of mV when the membrane conductance has its resting value. The effect on the membrane potential at other membrane conductances will be inversely proportional to the conductance. Note also that as in other types of IF neuron models, our neuron models do not actually generate action potential-like spikes but only identified moments of spikes, so “spiking” shown in all neuron simulations are represented graphically by assigning vertical spike-like lines at computed times of threshold crossing. The population “E-Aug-Late-HT” in Rybak et al. (2008) has been renamed to “E-Aug-Cough” in the base model for this simulation. >, neuron populations that relay perturbations of the network model. A fiber population composed of 100 fibers, each with a firing probability of 0.05 at each simulation time step and 100 type Ex_1 excitatory synaptic terminals (synaptic strength 0.03), represented cough receptor excitation. These fibers excited the Cough 2nd order neuron population. For abbreviations, see list of abbreviations and legend of Figure 1 in the main text*.

**Table 3:** **Synaptic parameters for the network model**.

Synapse name	Synapse type	Synapse equilibrium potential (mV)	Synapse time constant (ms)
Ex_1	Excitatory	115.0	1.5
Inh_4	Inhibitory	−25.0	1.5
Inh_7	Inhibitory	−25.0	2.0
Inh_10	Inhibitory	−25.0	1.5
Ex_13	Excitatory	115.0	1.5
Pre-ex_13	Inhibitory (pre-synaptic to Ex_13)	0.0	1.5
Ex_19	Excitatory	115.0	5.0
Inh_22	Inhibitory	−25.0	5.0
Inh_25	Inhibitory	−25.0	4.5
Ex_28	Excitatory	115.0	1.5
Pre-ex_28	Inhibitory (pre-synaptic to Ex_28)	−25.0	3.5

**Table 4:** **Connectivity for the network model modified from Poliaček et al. (2011)**.

Source population	Target population	Synaptic type	Conduction times		No. of terminals	Synaptic strength	Source pop. N	Target pop. N	Divergence	Mean no. of terminals	Convergence
			Min	Max	
I-Driver	I-Dec	ex_1	2	6	100	0.006	300	300	84.99 ± 3.14	1.18	84.99 ± 7.54
I-Driver	I-Aug	ex_1	2	6	100	0.01	300	300	84.93 ± 3.00	1.18	84.93 ± 7.73
I-Driver	I-Driver	ex_1	0	4	50	0.003	300	300	46.34 ± 1.76	1.08	46.34 ± 5.84
E-Dec-Phasic	I-Driver	inh_22	2	6	50	0.03	300	300	46.16 ± 1.77	1.08	46.16 ± 9.24
E-Dec-Phasic	E-Aug-early	inh_4	0	2	150	0.012	300	300	118.24 ± 4.01	1.27	118.24 ± 8.86
E-Dec-Phasic	E-Aug-late	inh_4	2	4	150	0.04	300	300	118.10 ± 3.94	1.27	118.10 ± 9.61
E-Dec-Phasic	VRC-IE	inh_4	0	2	50	0.1	300	99	39.33 ± 2.31	1.27	119.18 ± 7.22
E-Dec-Phasic	I-Dec	inh_4	0	2	200	0.2	300	300	146.07 ± 4.42	1.37	146.07 ± 8.71
+E-Dec-Phasic	I-Aug-BS	inh_4	2	6	100	0.15	300	300	85.21 ± 3.08	1.17	85.21 ± 7.27
E-Dec-Phasic	I-Aug	inh_4	2	6	50	0.1	300	300	46.19 ± 1.72	1.08	46.19 ± 5.43
E-Dec-Phasic	rostral IE-pons	ex_13	2	4	100	0.001	300	100	63.13 ± 2.98	1.58	189.39 ± 6.74
E-Dec-Phasic	caudal IE-pons	ex_13	2	4	100	0.001	300	100	63.23 ± 3.19	1.58	189.70 ± 9.56
+E-Dec-Phasic	I-Dec_2	inh_4	2	6	150	0.1	300	300	118.39 ± 4.42	1.27	118.39 ± 8.69
+E-Dec-Phasic	E-Aug (+)	inh_4	2	6	100	0.03	300	300	85.18 ± 2.99	1.17	85.18 ± 10.46
+E-Dec-Phasic	E-Aug-Cough (−)	inh_22	2	6	100	0.025	300	300	85.20 ± 3.05	1.17	85.20 ± 9.59
I-Dec	E-Aug-early	inh_7	2	6	115	0.5	300	300	95.67 ± 3.39	1.20	95.67 ± 7.14
I-Dec	E-Dec-Phasic	inh_25	2	6	200	0.2	300	300	145.80 ± 5.20	1.37	145.80 ± 9.31
I-Dec	I-Aug	inh_25	2	6	120	0.025	300	300	99.27 ± 3.48	1.21	99.27 ± 8.70
I-Dec	E-Aug-late	inh_7	2	6	115	0.5	300	300	95.71 ± 3.51	1.20	95.71 ± 7.84
I-Dec	VRC-IE	inh_25	0	4	33	0.025	300	99	28.15 ± 1.85	1.17	85.29 ± 6.01
I-Dec	E-Dec-Tonic	inh_25	2	6	100	0.001	300	300	84.90 ± 2.96	1.18	84.90 ± 9.43
I-Dec	ILM	ex_1	0	3	50	0.002	300	300	46.35 ± 1.71	1.08	46.35 ± 6.00
I-Dec	E-Aug-BS (+)	inh_7	0	4	100	0.05	300	300	85.44 ± 3.02	1.17	85.44 ± 9.96
I-Dec	EI-pons	ex_13	2	4	100	0.001	300	100	63.33 ± 3.16	1.58	190.00 ± 8.78
I-Dec	I-pons	ex_13	2	4	100	0.0005	300	100	63.50 ± 3.23	1.57	190.51 ± 8.03
I-Dec	I-pons	inh_7	2	4	100	0.0005	300	100	63.66 ± 3.21	1.57	190.98 ± 8.42
I-Dec	rostral IE-pons	inh_4	2	4	100	0.0001	300	100	63.55 ± 3.04	1.57	190.65 ± 8.05
I-Dec	caudal IE-pons	inh_7	2	4	100	0.0001	300	100	63.27 ± 3.06	1.58	189.80 ± 8.43
I-Dec	Lumbar	inh_7	0	4	100	0.1	300	210	79.70 ± 3.14	1.25	113.86 ± 8.97
I-Dec	E-Dec-pre-ELM	inh_4	2	6	200	0.06	300	300	146.12 ± 4.70	1.37	146.12 ± 8.41
+I-Dec	E-Aug (+)	inh_4	0	2	130	1.0	300	300	105.84 ± 3.65	1.23	105.84 ± 7.43
+I-Dec	ELM	inh_4	0	5	200	0.06	300	300	146.22 ± 4.96	1.37	146.22 ± 9.62
+I-Dec	Lumbar-HT	inh_7	2	6	100	0.1	300	70	53.32 ± 2.84	1.88	228.53 ± 6.63
+I-Dec	I-Dec	inh_25	2	6	140	0.0125	300	300	112.11 ± 3.97	1.25	112.11 ± 9.09
+I-Dec	E-Aug-Cough (−)	inh_7	2	6	115	0.16	300	300	95.71 ± 3.51	1.20	95.71 ± 7.84
I-Aug	I-Aug	ex_1	0	5	50	0.025	300	300	45.99 ± 1.77	1.09	45.99 ± 5.31
I-Aug	caudal IE-pons	inh_7	2	4	100	0.0001	300	100	63.43 ± 3.19	1.58	190.28 ± 9.05
I-Aug	I-Aug-BS	ex_1	2	6	100	0.06	300	300	85.20 ± 3.01	1.17	85.20 ± 7.27
I-Aug	ILM	ex_1	2	6	70	0.035	300	300	62.72 ± 2.32	1.12	62.72 ± 6.93
I-Aug	VRC-IE	ex_1	2	6	16	0.0015	300	99	14.82 ± 0.97	1.08	44.92 ± 6.80
I-Aug	I-pons	ex_13	2	4	100	0.0025	300	100	63.59 ± 2.94	1.57	190.76 ± 7.71
I-Aug	rostral IE-pons	inh_4	2	4	100	0.0001	300	100	63.43 ± 2.95	1.58	190.29 ± 8.57
+I-Aug	I-Dec_2	ex_28	2	6	200	0.01	300	300	146.05 ± 4.72	1.37	146.05 ± 9.65
E-Aug-early	E-Dec-Phasic	inh_4	2	6	110	0.007	300	300	92.32 ± 3.45	1.19	92.32 ± 7.95
E-Aug-early	I-Dec	inh_4	0	5	100	0.06	300	300	85.13 ± 2.98	1.17	85.13 ± 7.70
E-Aug-early	I-Aug	inh_4	2	6	100	0.135	300	300	85.44 ± 3.23	1.17	85.44 ± 7.76
E-Aug-early	VRC-IE	inh_4	0	2	24	0.05	300	99	21.46 ± 1.29	1.12	65.02 ± 6.96
E-Aug-early	I-Aug-BS	inh_4	0	2	150	0.001	300	300	118.31 ± 4.21	1.27	118.31 ± 7.57
E-Aug-early	E-Aug-late	inh_10	0	2	50	0.001	300	300	46.01 ± 1.81	1.09	46.01 ± 6.57
E-Aug-early	E-pons	ex_13	2	4	100	0.002	300	100	63.08 ± 2.85	1.59	189.25 ± 7.68
E-Aug-early	I-pons	inh_7	2	4	100	0.0005	300	100	63.08 ± 2.85	1.59	189.25 ± 7.68
+E-Aug-early	I-Dec_2	inh_4	2	6	150	0.08	300	300	118.31 ± 4.21	1.27	118.31 ± 7.57
+E-Aug-early	Phrenic	inh_4	0	2	150	0.001	300	210	107.57 ± 4.12	1.39	153.68 ± 8.34
+E-Aug-early	Phrenic-HT	inh_4	0	2	150	0.001	300	70	61.97 ± 2.40	2.42	265.57 ± 5.37
+E-Aug-early	ELM	inh_4	2	6	175	0.007	300	300	132.52 ± 4.21	1.32	132.52 ± 9.21
E-Aug-late	E-Aug-early	inh_10	0	2	200	0.04	300	300	145.91 ± 4.71	1.37	145.91 ± 9.07
E-Aug-late	I-Dec	inh_4	2	6	55	0.2	300	300	50.33 ± 1.87	1.09	50.33 ± 6.68
E-Aug-late	I-Aug	inh_4	2	6	100	0.1	300	300	85.27 ± 3.04	1.17	85.27 ± 7.41
E-Aug-late	E-Dec-Phasic	inh_4	2	6	120	0.015	300	300	98.86 ± 3.48	1.21	98.86 ± 9.67
E-Aug-late	I-Aug-BS	inh_4	2	6	150	0.06	300	300	118.14 ± 4.18	1.27	118.14 ± 8.44
E-Aug-late	VRC-IE	inh_4	0	2	24	0.02	300	99	21.32 ± 1.39	1.13	64.62 ± 7.03
E-Aug-late	E-Dec-Tonic	inh_4	0	2	100	0.05	300	300	85.18 ± 3.01	1.17	85.18 ± 6.53
E-Aug-late	E-Dec-pre-ELM	inh_22	2	6	115	0.05	300	300	95.69 ± 3.35	1.20	95.69 ± 7.07
E-Aug-late	ELM	inh_7	2	6	200	0.1	300	300	145.91 ± 4.71	1.37	145.91 ± 9.07
+E-Aug-late	I-Dec_2	inh_4	2	6	150	0.075	300	300	118.14 ± 4.18	1.27	118.14 ± 8.44
+E-Aug-late	Phrenic	inh_4	4	8	150	0.06	300	210	107.09 ± 3.99	1.40	152.98 ± 7.22
+E-Aug-late	Phrenic-HT	inh_4	0	2	150	0.06	300	70	61.83 ± 2.37	2.43	265.00 ± 5.23
+E-Aug-late	ILM	inh_4	0	2	115	0.04	300	300	95.59 ± 3.32	1.20	95.59 ± 8.06
VRC-IE	I-Dec	inh_7	0	4	200	0.035	99	300	146.65 ± 4.99	1.36	48.39 ± 5.40
Raphé 8	Raphé 29	ex_1	0	3	50	0.0125	100	100	39.73 ± 2.28	1.26	39.73 ± 5.01
Raphé 28	Raphé 30	ex_1	0	3	50	0.0125	100	100	39.51 ± 2.47	1.27	39.51 ± 4.93
+I-Aug-BS	Phrenic-HT	ex_1	3	6	18	0.05	300	70	15.99 ± 1.24	1.13	68.51 ± 7.54
+I-Aug-BS	Phrenic	ex_1	3	6	50	0.05	300	210	44.95 ± 2.01	1.11	64.21 ± 6.33
NRM-BötC	rostral IE-pons	inh_7	0	1	100	0.002	300	100	63.41 ± 3.22	1.58	190.23 ± 7.92
NRM-BötC	caudal IE-pons	inh_7	0	1	100	0.002	300	100	63.37 ± 3.02	1.58	190.12 ± 7.75
NRM-BötC	I-pons	ex_13	0	1	100	0.002	300	100	63.25 ± 3.00	1.58	189.74 ± 8.31
+E-Aug-Cough (−)	E-Aug (+)	inh_22	2	6	100	0.05	300	300	85.39 ± 3.06	1.17	85.39 ± 7.75
E-Aug-Cough (−)	E-Aug-BS (+)	inh_22	2	6	100	0.5	300	300	85.34 ± 3.04	1.17	85.34 ± 7.22
+E-Aug-Cough (−)	I-Dec_2	inh_22	0	3	200	0.05	300	300	145.65 ± 4.67	1.37	145.65 ± 8.84
E-Aug-Cough (−)	E-Dec-pre-ELM	inh_4	2	6	100	0.1	300	300	85.22 ± 3.02	1.17	85.22 ± 7.02
+E-Aug-Cough (−)	I-Aug-BS	inh_4	0	3	200	0.025	300	300	145.65 ± 4.67	1.37	145.65 ± 8.84
+E-Aug-Cough (−)	ILM	inh_4	0	3	200	0.025	300	300	146.45 ± 4.63	1.37	146.45 ± 8.96
Lung PSRs	Pump (+)	ex_1	0	3	75	0.015	300	300	66.50 ± 2.43	1.13	66.50 ± 6.72
Lung PSRs	Pump (−)	ex_1	0	3	50	0.015	300	300	46.23 ± 1.78	1.08	46.23 ± 9.43
caudal IE-pons	I-Driver	ex_1	0	5	100	0.001	100	300	85.68 ± 2.78	1.17	28.56 ± 4.45
Pump (–)	E-pons	pre-ex_13	0	4	100	0.99	300	100	63.19 ± 2.88	1.58	189.58 ± 7.28
+Pump (–)	I-Dec_2	inh_4	0	2	25	0.0035	300	300	24.04 ± 0.88	1.04	24.04 ± 5.11
Pump (–)	I-Dec	inh_4	0	2	25	0.0035	300	300	23.98 ± 0.90	1.04	23.98 ± 5.97
Pump (–)	I-pons	pre-ex_13	0	4	100	0.99	300	100	63.53 ± 2.94	1.57	190.58 ± 7.02
Pump (–)	EI-pons	pre-ex_13	2	4	100	0.99	300	100	63.55 ± 2.95	1.57	190.64 ± 7.44
Pump (–)	Lung Def_1s	inh_4	0	4	100	0.02	300	300	85.22 ± 3.09	1.17	85.22 ± 7.46
Pump (–)	rostral IE-pons	pre-ex_13	0	4	100	0.99	300	100	63.63 ± 3.02	1.57	190.88 ± 9.09
Pump (–)	caudal IE-pons	pre-ex_13	0	4	100	0.99	300	100	63.63 ± 3.02	1.57	190.88 ± 9.09
Pump (+)	E-Dec-Phasic	ex_1	0	2	100	0.01	300	300	85.47 ± 2.95	1.17	85.47 ± 8.14
Pump (+)	VRC-IE	ex_1	2	6	100	0.01	300	99	63.10 ± 2.99	1.58	191.20 ± 11.20
Pump (+)	I-Aug	ex_1	0	2	25	0.0	300	300	24.07 ± 0.91	1.04	24.07 ± 4.15
Pump (+)	E-Dec-T	ex_1	0	2	100	0.002	300	300	85.12 ± 3.16	1.17	85.12 ± 6.94
+Pump (+)	I-Dec_2	ex_1	2	6	100	0.005	300	300	85.20 ± 3.05	1.17	85.20 ± 9.59
E-Dec-T	Raphé 29	inh_4	0	3	100	0.001	300	100	63.59 ± 3.05	1.57	190.76 ± 7.20
E-Dec-T	I-Aug-BS	inh_4	2	6	100	0.03	300	300	84.99 ± 3.14	1.18	84.99 ± 7.54
E-Dec-T	rostral IE-pons	ex_13	2	4	100	0.001	300	100	63.26 ± 3.11	1.58	189.77 ± 11.78
E-Dec-T	I-pons	ex_13	2	4	100	0.0005	300	100	63.33 ± 3.02	1.58	189.98 ± 9.72
E-Dec-T	I-pons	inh_7	2	4	100	0.0005	300	100	63.20 ± 3.13	1.58	189.60 ± 7.79
E-Dec-T	rostral IE-pons	inh_4	2	4	100	0.0005	300	100	63.57 ± 3.16	1.57	190.70 ± 7.57
E-Dec-T	caudal IE-pons	ex_13	2	4	100	0.001	300	100	63.20 ± 3.18	1.58	189.61 ± 7.72
E-Dec-T	caudal IE-pons	inh_7	2	4	100	0.0005	300	100	63.40 ± 3.17	1.58	190.20 ± 9.74
E-Dec-T	ELM	inh_4	0	4	100	0.04	300	300	85.04 ± 3.30	1.18	85.04 ± 8.24
E-Dec-T	I-Aug	inh_4	2	6	100	0.0075	300	300	85.12 ± 3.35	1.17	85.12 ± 8.17
+E-Dec-T	I-Dec_2	pre-ex_28	2	6	100	0.2	300	300	84.99 ± 3.14	1.18	84.99 ± 7.54
rostral IE-pons	EI-pons	inh_4	2	4	100	0.03	100	100	63.79 ± 3.24	1.57	63.79 ± 4.98
rostral IE-pons	VRC-IE	ex_1	0	1	100	0.001	100	99	62.90 ± 3.28	1.59	63.54 ± 4.65
rostral IE-pons	E-Dec-Phasic	ex_1	0	5	100	0.02	100	300	85.06 ± 2.76	1.18	28.35 ± 4.15
EI-pons	rostral IE-pons	ex_1	2	4	100	0.002	100	100	63.47 ± 3.20	1.58	63.47 ± 4.78
EI-pons	caudal IE-pons	ex_1	2	4	100	0.002	100	100	63.36 ± 3.47	1.58	63.36 ± 4.41
EI-pons	VRC-IE	ex_1	0	4	50	0.0003	100	99	39.46 ± 2.35	1.27	39.86 ± 5.11
EI-pons	E-Dec-T	ex_1	0	4	100	0.01	100	300	85.16 ± 3.33	1.17	28.39 ± 5.10
E-Dec-pre-ELM	ELM	ex_19	2	6	250	0.0125	300	300	169.56 ± 5.08	1.47	169.56 ± 7.81
Def 2nd (−)	E-Dec-Phasic	inh_4	2	6	100	0.04	300	300	85.17 ± 3.11	1.17	85.17 ± 9.16
+E-Aug (+)	E-Aug-BS (+)	ex_19	2	6	100	0.02	300	300	85.16 ± 2.97	1.17	85.16 ± 7.43
Raphé 8	Raphé 31	inh_4	0	3	50	0.005	100	100	39.38 ± 2.09	1.27	39.38 ± 6.21
Raphé 8	Raphé 32	inh_4	0	3	50	0.005	100	100	39.51 ± 2.47	1.27	39.51 ± 4.93
Raphé 8	E-Aug-BS (+)	inh_22	0	3	400	0.0	100	300	221.45 ± 4.77	1.81	73.82 ± 4.47
Raphé 29	Raphé 30	ex_1	0	3	50	0.01	100	100	39.51 ± 2.47	1.27	39.51 ± 4.93
Raphé 29	E-Dec-T	ex_19	0	3	100	0.15	100	300	84.74 ± 3.12	1.18	28.25 ± 4.23
Raphé 29	E-Dec-Phasic	ex_19	0	3	100	0.2	100	300	84.74 ± 3.12	1.18	28.25 ± 4.23
Raphé 30	Raphé 29	inh_4	0	3	50	0.01	100	100	39.51 ± 2.47	1.27	39.51 ± 4.93
Raphé 32	Raphé 31	inh_4	0	3	50	0.005	100	100	39.51 ± 2.47	1.27	39.51 ± 4.93
Raphé 32	E-Dec-Tonic	inh_22	0	3	100	0.01	100	300	85.28 ± 3.13	1.17	28.43 ± 4.65
Raphé 32	E-Dec-Phasic	inh_22	0	3	100	0.01	100	300	84.74 ± 3.12	1.18	28.25 ± 4.23
Raphé 32	E-Dec-pre-ELM	inh_22	0	3	100	0.01	100	300	84.74 ± 2.97	1.18	28.25 ± 4.67
>+Cough 2nd order (+)	I-Aug-BS	ex_1	2	6	100	0.02	100	300	85.25 ± 2.83	1.17	28.42 ± 5.15
>Cough 2nd order (+)	I-Aug	ex_1	2	6	100	0.0045	100	300	85.25 ± 2.83	1.17	28.42 ± 5.15
>Cough 2nd order (+)	I-Dec	ex_1	2	6	100	0.0045	100	300	85.27 ± 2.89	1.17	28.42 ± 4.68
>+Cough 2nd order (+)	I-Dec_2	ex_1	2	6	100	0.05	100	300	85.54 ± 3.07	1.17	28.51 ± 4.72
>Cough 2nd order (+)	E-Aug-late	ex_1	2	6	100	0.005	100	300	85.25 ± 2.83	1.17	28.42 ± 5.15
>Cough 2nd order (+)	E-Aug-early	ex_1	0	3	100	0.01	100	300	85.07 ± 3.05	1.18	28.36 ± 4.19
>Cough 2nd order (+)	VRC-IE	inh_4	0	3	100	0.2	100	99	63.13 ± 3.05	1.58	63.77 ± 5.20
>Cough 2nd order (+)	caudal IE-pons	ex_1	0	3	100	0.001	100	100	63.59 ± 3.21	1.57	63.59 ± 5.84
>Cough 2nd order (+)	rostral IE-pons	ex_1	0	3	100	0.001	100	100	63.59 ± 3.21	1.57	63.59 ± 5.84
>Cough 2nd order (+)	I-pons	ex_1	0	3	100	0.001	100	100	63.59 ± 3.21	1.57	63.59 ± 5.84
>Cough 2nd order (+)	E-pons	ex_1	2	6	100	0.001	100	100	63.59 ± 3.21	1.57	63.59 ± 5.84
>Cough 2nd order (+)	EI-pons	ex_1	0	3	100	0.001	100	100	63.59 ± 3.21	1.57	63.59 ± 5.84
>Cough 2nd order (+)	E-Dec-pre-ELM	ex_19	2	6	100	0.004	100	300	85.14 ± 3.06	1.17	28.38 ± 3.97
>+Cough 2nd order (+)	E-Aug (+)	ex_1	2	6	100	0.05	100	300	85.25 ± 2.83	1.17	28.42 ± 5.15
>+Cough 2nd order (+)	E-Aug-Cough (−)	ex_1	2	6	100	0.04	100	300	85.25 ± 2.83	1.17	28.42 ± 5.15
>+Cough 2nd order (+)	ILM	ex_1	2	6	100	0.001	100	300	84.92 ± 3.23	1.18	28.31 ± 4.63
E-pons	rostral IE-pons	inh_4	2	4	100	0.0001	100	100	63.17 ± 3.15	1.58	63.17 ± 5.22
E-pons	caudal IE-pons	inh_4	2	4	100	0.0001	100	100	63.47 ± 3.13	1.58	63.47 ± 5.60
E-pons	I-Dec	inh_4	0	1	100	0.008	100	300	85.14 ± 3.03	1.17	28.38 ± 4.11
NRM-pons	I-pons	ex_1	0	4	100	0.015	100	100	63.26 ± 3.28	1.58	63.26 ± 4.40
NRM-pons	I-pons	inh_4	0	4	100	0.05	100	100	63.62 ± 3.11	1.57	63.62 ± 4.61
NRM-pons	I-Driver	ex_1	2	6	100	0.11	100	300	85.10 ± 3.00	1.18	28.37 ± 4.51
NRM-pons	VRC-IE	ex_1	0	1	100	0.01	100	99	63.02 ± 2.53	1.59	63.66 ± 4.67
NRM-pons	I-Aug	ex_1	0	1	100	0.01	100	300	85.21 ± 2.94	1.17	28.40 ± 4.81
NRM-pons	E-Aug-early	ex_1	0	4	100	0.025	100	300	85.82 ± 3.10	1.17	28.61 ± 4.20
NRM-pons	E-Aug-late	ex_1	0	4	50	0.003	100	300	45.82 ± 1.89	1.09	15.27 ± 3.67
NRM-pons	E-Dec-Phasic	ex_1	0	1	100	0.01	100	300	84.87 ± 3.22	1.18	28.29 ± 4.18
NRM-pons	E-Dec-Tonic	ex_1	0	1	100	0.1	100	300	85.35 ± 3.04	1.17	28.45 ± 4.06
NRM-pons	NRM-BötC	inh_4	0	1	100	0.001	100	300	85.11 ± 2.96	1.17	28.37 ± 5.07
E-Aug-BS (+)	Lumbar	ex_1	6	10	25	0.03	300	210	23.59 ± 1.14	1.06	33.70 ± 5.45
+E-Aug-BS (+)	Lumbar-HT	ex_1	3	6	10	0.05	300	70	9.34 ± 0.75	1.07	40.03 ± 3.18
I-pons	rostral IE-pons	ex_1	0	4	100	0.005	100	100	62.93 ± 2.89	1.59	62.93 ± 5.56
I-pons	VRC-IE	ex_1	0	5	100	0.005	100	99	63.61 ± 3.41	1.57	64.25 ± 4.84
I-pons	I-Aug	ex_1	0	4	50	0.005	100	300	46.17 ± 1.67	1.08	15.39 ± 3.39
I-pons	caudal IE-pons	ex_1	0	4	100	0.005	100	100	63.67 ± 2.85	1.57	63.67 ± 4.57
+I-Dec_2	E-Dec-Tonic	inh_4	2	6	125	0.1	300	300	102.25 ± 3.60	1.22	102.25 ± 7.81
+I-Dec_2	E-Dec-pre-ELM	inh_4	2	6	100	0.01	300	300	85.11 ± 3.10	1.17	85.11 ± 7.52
+I-Dec_2	E-Aug-BS (+)	inh_4	2	6	50	0.00005	300	300	46.04 ± 1.73	1.09	46.04 ± 8.13
+I-Dec_2	ELM	inh_4	2	6	100	0.02	300	300	85.61 ± 3.37	1.17	85.61 ± 7.12
+I-Dec_2	E-Aug (+)	inh_4	2	6	100	0.02	300	300	85.19 ± 3.09	1.17	85.19 ± 8.08
+Lung Def_1s	Def 2nd (−)	ex_1	0	3	35	0.03	300	300	33.04 ± 1.38	1.06	33.04 ± 6.68
+Lung DIS_1s	ELM	ex_1	0	3	100	0.09	300	300	84.97 ± 2.93	1.18	84.97 ± 7.40

*+, Connection added to the network in Poliaček et al. (2011). >, Connection relaying a perturbation to the network model. Connections between individual neurons were made according to a sequence of pseudorandom numbers calculated from a unique seed number for each source-to-target connection. Targets were chosen with replacement. This table includes the means ± SD of the number of neurons in each target population innervated by each source neuron in each population. Corresponding values are also shown for source neurons that innervated each target neuron in each population. These data indicate the extent of divergence and convergence, respectively. Most neurons in each source population made a single terminal connection with each target neuron. *Mean No. of Terminals*, the mean number of terminals from each source neuron innervating each target neuron. The efficacy of connections between populations of neurons was influenced by the change in conductance associated with each action potential at a synapse (*Synaptic Strength*) and the number of terminals for each axon. *Synaptic types* were distinguished by their equilibrium potentials and time constants. The time constant of some synapses was slightly longer than others because troughs in cross-correlograms from which the particular synaptic connections were inferred tended to have longer durations. 11 types of synapses were used in the simulation (see Table 3). If the value of the presynaptic modulatory strength parameter (*Synaptic Strength*) was <1.0, the strength of the connection it modulated was reduced to the product of the presynaptic Synaptic Strength parameter and target synapse conductance. If the presynaptic Synaptic Strength parameter was >1.0, the amount by which it was greater than 1 was added to its target synapse’s conductance. *Minimum and maximum conduction times* are expressed in 0.5 ms simulation clock ticks for each source-to-target axon population. *No. of Terminals*, number of terminals from source neuron*.

#### Linking the neural network and biomechanical models

The diaphragm received input from two phrenic motor neuron populations (PHR, PHR-HT) with different threshold ranges to generate motor unit diversity and facilitate an ordered recruitment during increased inspiratory drive. Diaphragm activation was based on the mean instantaneous firing rates (*P*, *P*_1_), of the two populations by the expression (0.3*P* + 0.7*P*_1_)/*X* where *X* is the firing rate for maximum diaphragm activation; values of 50–200 spikes/s were used (Nail et al., 1972) to approximate the plot for diaphragm activation in Figure 1 of Mantilla and Sieck (2011). Similarly, two lumbar motor neuron populations (LUM, LUM-HT) activated the abdominal muscle with *X* set to 80. Inspiratory laryngeal motor (ILM) and expiratory laryngeal motor (ELM) neuron populations regulated laryngeal resistance over a range between fully open (+1) and fully closed (−1), inclusively.

Lung afferent populations were regulated by injected currents defined at each simulation step by evaluation of expressions that included model lung volume. Pulmonary stretch receptors (PSRs) became more active with increasing lung volume *V* (membrane bias = 0.5*V* mV/%VC) and mediated the Hering–Breuer reflex. Deflation-sensitive lung receptors were also implemented (Paintal, 1955; Luck, 1970; Wei and Shen, 1985; Iscoe and Gordon, 1992; Bergren and Peterson, 1993; Matsumoto et al., 2002; Yu et al., 2003). A low threshold population (Def_1, membrane bias = − 0.225(*V* − 70) mV/%VC) and its afferent pathway introduced in Poliaček et al. (2011) was used to represent a class of possible network mechanisms for generating an inhibitory bias on E-Dec neurons. Simulated “vagotomy” (elimination of the effects of lung afferents) in the present model removed this inhibition, contributing to the observed prolongation of the expiratory phase (Te increased from 2.76 s to 3.15 s (*p*-value = 0.0004, two-sided *t*-test). Vagotomy also removed the influence of the PSRs and increased inspiratory phase duration (Ti) from 1.94 s to 2.61 s, (*p*-value = 4 × 10^−7^; see references and discussion in Dick et al., 2008). A higher threshold “distortion” (Dis_1) receptor population (cf. Iscoe and Gordon, 1992) excited the ELM population when lung volume was below FRC (membrane bias = −1.75(*V* − 10) mV/%VC if *V *< 10, 0 otherwise). We note that the synaptic strength and firing rates of this speculative Dis_1 population, added for development purposes in other work (Hutchison and Lindsey, 2009), resulted in negligible modulation of ELM population activity under the conditions of the present study (see “Influence of Some Added Network Connections”).

#### Additional enhancements to the current network model

The “I-Dec_2” population was added to provide a second inhibitory VRC inspiratory neuron population for tuning inspiratory drive as proposed in Ott et al. (2012) for central chemoreceptor modulation of breathing. In some previous models (Rybak et al., 2008; Poliaček et al., 2011), the “E-Aug-late” population inhibited numerous target populations, but also served to excite the VRG bulbospinal E-Aug-BS (+) population that drives expiratory lumbar motor neurons. A new “E-Aug (+)” population was added to facilitate differential regulation of the lumbar motor neurons and expiratory drive modulation as proposed in the literature (Iscoe, 1998; Shannon et al., 2004; Molkov et al., 2010). Other parameters were adjusted and populations added in anticipation of linking the network model to the biomechanical model derived from data from human subjects. In the antecedent model (Poliaček et al., 2011), the I-Aug-BS population output served both a premotor function and represented the “phrenic” output. The inhibitory connections from the VRC-IE population to the I-Aug-BS population were eliminated in the new model; E-Dec-P inhibition of the I-Aug-BS population was retained. The resulting eupneic respiratory cycle frequency (12.7 cycles/min) was within the range for resting breathing in the human adult (A.D.A.M. Medical Encyclopedia, 2012).

#### Eupneic motor pattern and “baseline” cough

**Figure 2:**
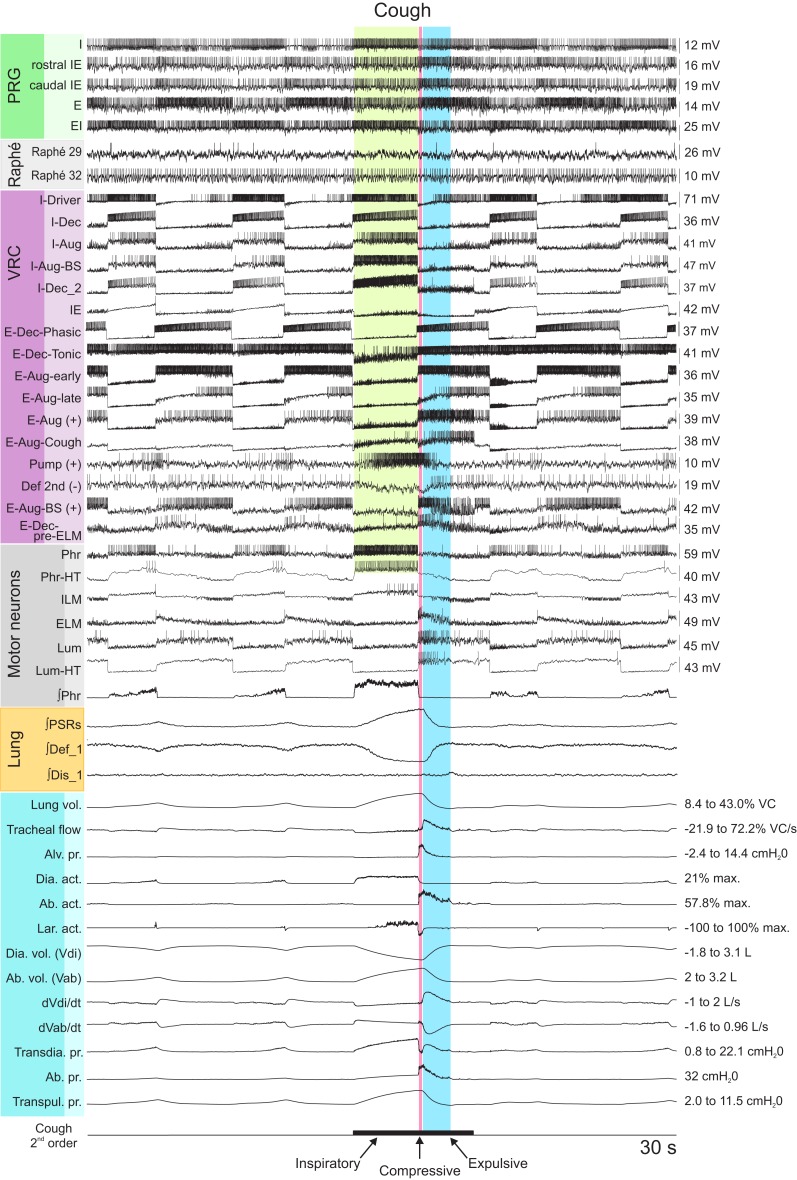
**Simulated eupneic respiratory cycles and an evoked cough motor pattern (inspiratory, compressive, and expulsive phases respectively labeled and highlighted by colored columns)**. The top 29 traces show membrane potentials and discharge patterns of individual respiratory neurons from a subset of the simulated populations as indicated by the labels on the left, arranged by region (PRG, raphé, VRC) or type (Motor neuron populations). The “integrated” phrenic trace represents the threshold crossing activity of the “PHR” population summed over 60 ms windows and indicates the inspiratory and expiratory phases of the respiratory cycle. Similarly, integrated traces from three lung afferent populations are plotted below the motor neuron records. (PSR, pulmonary stretch receptors) The 13 traces below those from the afferents show, in order from top to bottom: 1: lung volume (%VC, relative to RV); 2: tracheal flow (%VC/s, expiration positive (up)); 3: alveolar pressure (cmH_2_O); 4–6: diaphragm activation, abdominal muscle activation, and net laryngeal muscle activation (dimensionless ratios to maximums); 7: diaphragm volume (L); 8: abdominal volume (L); 9: derivative of diaphragm volume (L/s); 10: derivative of abdominal volume (L/s); 11–13: transdiaphragmatic, abdominal, and transpulmonary pressures (cmH_2_O). The bottom trace indicates the duration of a simulated cough stimulus. A fiber population composed of 100 fibers, each with a firing probability of 0.05 at each simulation time step and 100 type Ex_1 excitatory synaptic terminals (synaptic strength 0.03), represented cough receptor excitation. These fibers excited the Cough 2nd order neuron population (Figure 1); see Table 2 for properties of this population and Table 4 for details of connections with other populations. See text for further discussion.

The joint neuromechanical model generated a eupneic motor pattern and an evoked cough. Figure 2 shows membrane potential records from simulated neurons in representative PRG, raphé, and VRC neuron populations and the six types of motor neurons. The “IF” neuron populations do not generate action potential-like spikes; instances of threshold crossings are indicated graphically by corresponding vertical spike-like lines. Additional traces include integrated population activity of the three lung afferent populations and biomechanical system metrics, including lung volume, tracheal flow, and alveolar pressure. The three phases of the cough cycle (Bolser et al., 2003) are highlighted.

The inspiratory phase of the cough was characterized by increased activation of the diaphragm and enlargement of upper airway via activation of the ILM (abductor) motor neuron population, resulting in an increased lung volume (43% VC), inspiratory flow, and transdiaphragmatic and transpulmonary pressures. The subsequent compressive phase included activation of the ELM (adductor) motor neurons with transient laryngeal closure, together with activation of lumbar motor neurons and abdominal expiratory muscles. During this phase, tracheal airflow stopped and there was an increase in alveolar and abdominal pressure. In the following expulsive phase, high air flow velocity (72.2% VC/s) resulted from the opening of the larynx during continued abdominal muscle activation.

#### Cough behavior with changes in inspiratory drive

Two series of simulations with complementary perturbations of cough inspiratory drive were made to assess model behavior during the phases of cough. Different sets of random number seeds were used for each simulation to generate variability in model output by altering the thresholds of individual neurons in each population and the convergent and divergent connectivity patterns among populations within ranges defined by the initial baseline parameter settings.

**Figure 3:**
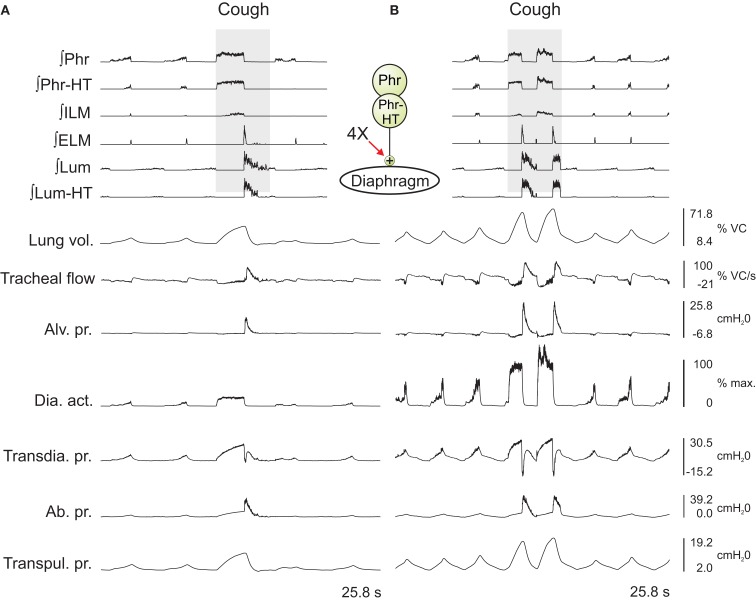
**Integrated traces of motor neuron population activities (top 6 traces, population labels on left) and biomechanical model outputs (labels on left; see legends of Figures 1 and 2 for definitions) during eupneic and “baseline” cough motor patterns before (A) and after (B) increasing the gain of diaphragm activation by a factor of 4 (see center schematic inset); otherwise, cough stimulus parameters as in Figure 2**. Scales on right are for **(A,B)**. See text for further details.

In the first series, the activation strengths for the connections between phrenic motor neuron populations and the diaphragm were increased by factors of 2 and 4. Figure 3 shows the integrated activity of each motor neuron population together with lung volume, tracheal flow, alveolar pressure, and abdominal pressure for baseline conditions (left) and a trial with four times the baseline activation strength (right).

**Figure 4:**
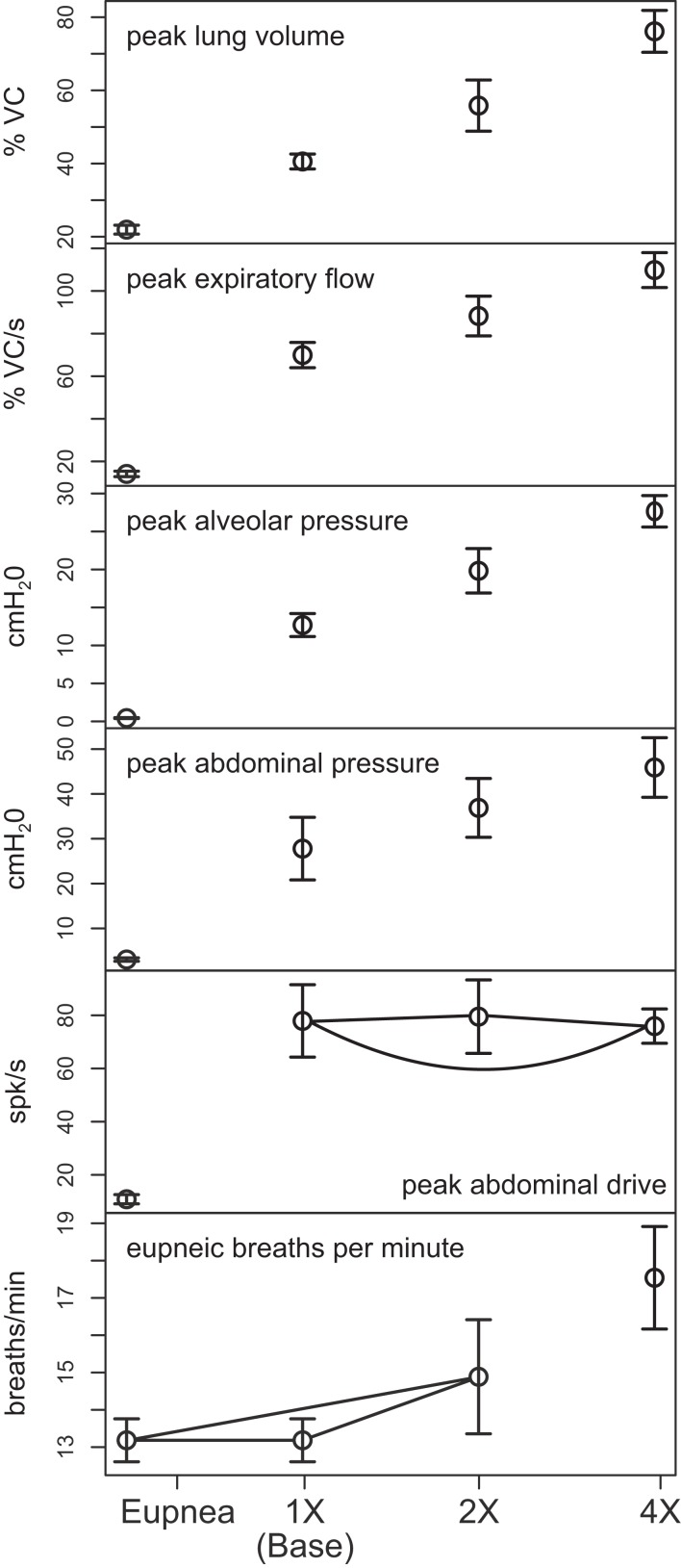
**Means of peak values (±95% confidence limits) of (from top) lung volume, expiratory tracheal flow, alveolar pressure, abdominal pressure, and abdominal drive together with respiratory cycle frequencies during pre-cough eupneic intervals (bottom) measured during model simulations of baseline cough (1×) and two conditions of increased phrenic-to-diaphragm activation gain (2×, 4×)**. Pairs of symbols connected by a line indicate no significant difference.

Outputs from four trials for each amplified condition were compared with each other and with the baseline results. Figure 4 shows the means (±95% confidence limits) of selected biomechanical outputs measured during baseline cough (1×) and the two conditions of increased activation gain (2×, 4×). Pairs of symbols connected by a line indicate no significant difference. Successively larger peak expiratory flow rates and abdominal pressures were respectively associated with greater lung volumes during preceding inspiratory phases of the evoked coughs, even though abdominal drive did not change. This result established that differences in flow with the generated changes in inspiratory (operating) volumes were the consequence of the modeled biomechanical system.

Mean respiratory cycle frequencies measured during pre-cough eupneic intervals for each condition were also evaluated. The respiratory frequency increased with the highest (4×) inspiratory drive, a change associated with changes in feedback from lung afferents under the “closed loop” conditions evaluated.

**Figure 5:**
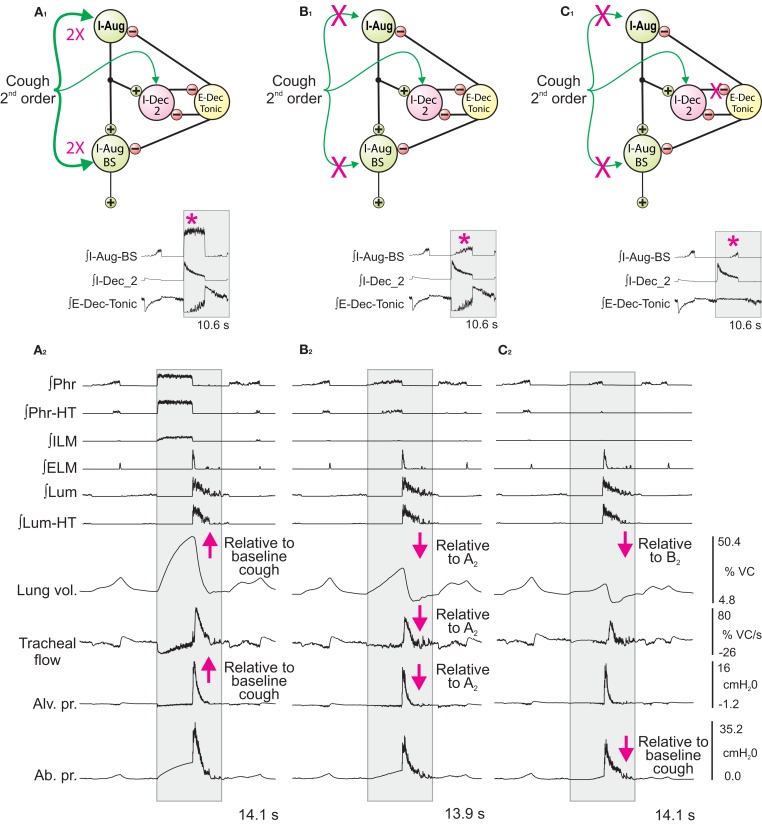
**Network perturbations that selectively alter cough inspiratory drive change lung volume and also influence tracheal flow and abdominal pressure during the subsequent expiratory phase of cough**. Each panel (**A_1_–C_1_**) shows a schematic of a subset of the model network and changes in synaptic strengths during simulated cough stimulation relative to the baseline conditions represented in and described for Figure 3A. Corresponding panels (**A_2_–C_2_**) show integrated traces of motor neuron population activities and biomechanical model outputs (left labels) for the respective perturbations of inspiratory drive. Arrows mark changes. See text for further details.

**Figure 6:**
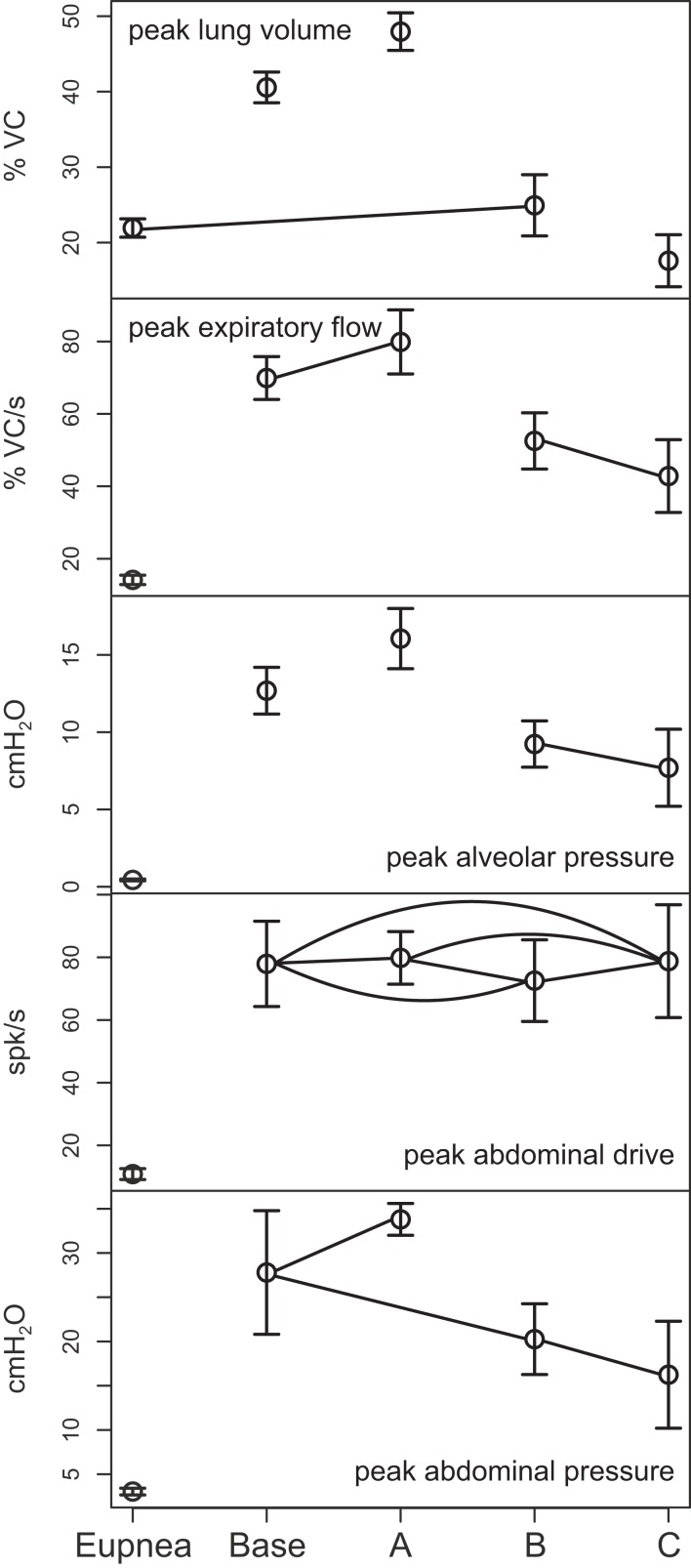
**Means of peak values (±95% confidence limits) of (from top) lung volume, expiratory tracheal flow, alveolar pressure, abdominal drive, and abdominal pressure measured under conditions of pre-cough eupnea (Eupnea), baseline cough (Base), doubling of the synaptic strength between Cough 2nd order and the I-Aug and I-Aug-BS populations (A), elimination of Cough 2nd order excitation of the I-Aug and I-Aug-BS populations (B), and additionally, the subsequent elimination of I-Dec_2 neuron inhibition of E-Dec-Tonic neurons *during* the inspiratory phase of the evoked cough cycle (C)**. Pairs of symbols connected by a line indicate no significant difference.

In the second series of simulations, inspiratory drive was altered only during the cough cycle by changing synaptic strengths of Cough 2nd order neuron inputs to selected model populations. The top panels in Figures 5A_1–C_1__ show schematics of a subset of the model network and sites where synaptic strengths were changed relative to the baseline conditions represented in and described for Figure 3A. Corresponding panels in Figures 5A_2–C_2__ show integrated traces of motor neuron population activities and biomechanical model outputs for the respective perturbations; arrows highlight significant changes in the indicated metrics (further enumerated in Figure 6).

First (Figure 5A), synaptic strengths from the Cough 2nd order population to the I-Aug and I-Aug-BS populations were doubled. The highlighted segment of the inset (Figure 5A_1_) shows integrated traces for the I-Aug-BS, I-Dec_2, and E-Dec-Tonic populations during a eupneic cycle and the subsequent evoked cough. I-Aug-BS activity increased under this condition (asterisk).

Next (Figure 5B), cough inspiratory drive was decreased relative to baseline by deletion (synaptic strength = 0.0) of the excitatory connections between the Cough 2nd order population and both the I-Aug and I-Aug-BS populations. The excitation of the I-Dec_2 population by 2nd order cough neurons remained, partially suppressing the recurrent inhibition of the I-Dec_2 and two I-Aug populations. The highlighted segment of the inset (Figure 5B_1_) shows reduced I-Aug-BS activity during the evoked cough under this condition (asterisk).

The third perturbation further reduced cough inspiratory drive by also transiently blocking I-Dec_2 neuron inhibition of the E-Dec-Tonic population during the cough cycle. The elimination of this influence resulted in increased E-Dec-Tonic inhibition of the I-Aug and I-Aug-BS populations during the cough (asterisk in highlighted segment of Figure 5C_1_).

Figure 6 plots (from the top) the means of peak values (±95% confidence limits) for lung volume, expiratory tracheal flow, alveolar pressure, abdominal drive, and abdominal pressure measured under conditions of pre-cough eupnea (Eupnea), baseline cough (Base), and the three conditions represented in Figure 5. The differences in peak lung volumes during cough under the three conditions (A–C) confirm functional roles for both the excitatory and disinhibitory influences on inspiratory drive in the model. Deletion of the excitatory component of cough inspiratory drive (B) caused peak expiratory tracheal flow to decrease relative to the previous baseline and enhanced coughs (Base, A). However, peak abdominal drive and abdominal pressure did not change. A further reduction in peak lung volume to levels below eupneic control due to transiently increased E-Dec-Tonic inhibition of inspiratory drive (C) during cough resulted in no further change in expiratory flow, although peak abdominal pressure was reduced relative to baseline cough values.

**Figure 7:**
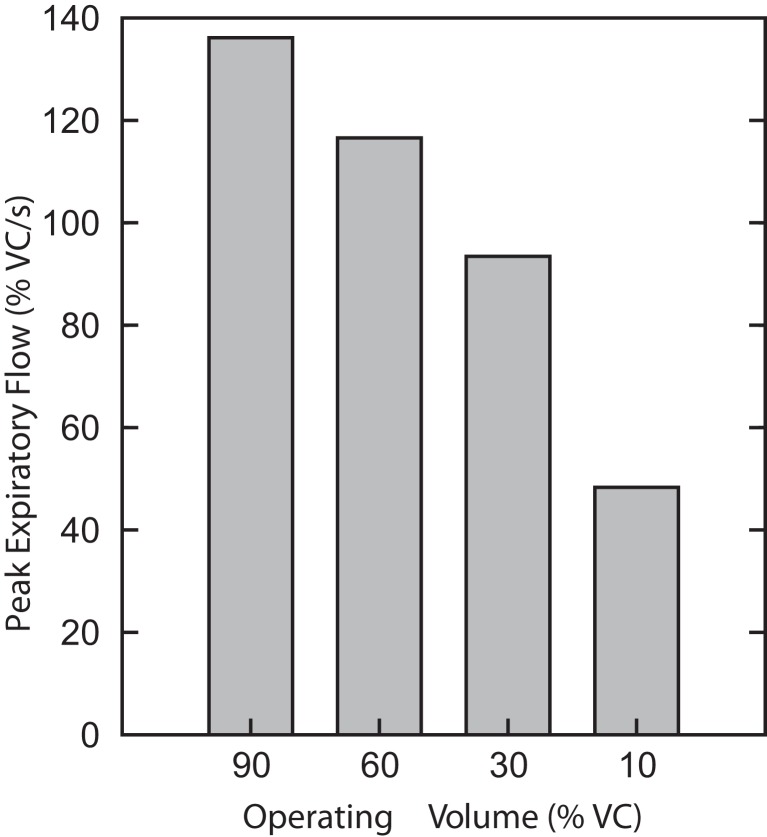
**Peak expiratory flow in four coughs simulated with the isolated biomechanical model at different operating volumes but equal peak abdominal pressure of 26.5 cmH_2_O**. There are no error bars because these are runs of the deterministic model.

A third series of simulations was done with the isolated biomechanical model. Figure 7 plots the peak expiratory flow in four coughs simulated at different operating volumes but equal peak abdominal pressure of 26.5 cmH_2_O. In each cough, the diaphragm and abdominal activations were first controlled to produce the desired operating volume, then the laryngeal muscles were controlled to close the airway, then the abdominal activation was controlled to produce an abdominal pressure of 26.5 cmH_2_O, and finally the laryngeal muscles were controlled to open the airway. Note that no statistics were done on these runs because the biomechanical model is deterministic. As in the first series of simulations, successively larger peak expiratory flow rates were associated with greater lung volumes during preceding inspiratory phases of the simulated cough, but unlike the first series, the peak abdominal pressure was the same in each cough. This result established that differences in flow with the generated changes in inspiratory (operating) volumes were not entirely due to the differences in pressure seen in the first series.

#### Comparisons with behaviors of antecedent models

Table 5 shows means of inspiratory and expiratory phase durations during eupnea and cough and peak firing rates of motor neuron populations common to the present model and those described in Rybak et al. (2008) and Poliaček et al. (2011). The current model has lower firing rates, similar to those observed *in vivo* (Iscoe, 1998; Mantilla and Sieck, 2011), and longer respiratory phase durations; inspiratory phase durations are also more variable (see coefficients of variation, Table 5).

**Table 5:** **Comparison with previous models**.

Variable	Unit	Rybak			Poliaček					No speculative				Current	
		Mean	*p*	CV	*p*	Mean	*p*	CV	*p*	Mean	*p*	CV	*p*	Mean	CV
**EUPNEA**															
ELM	spk/s	170	0.00*	0.10	0.00*	231	0.00*	0.08	0.00*	19	1.00	0.23	1.00	19	0.23
ILM	spk/s	83	0.00*	0.06	0.00*	49	0.00*	0.07	0.00*	26	0.62	0.29	1.00	19	0.47
LUMBAR	spk/s	24	0.00*	0.05	0.07	135	0.00*	0.08	1.00	10	1.00	0.08	1.00	11	0.08
PHRENIC	spk/s	109	0.00*	0.10	1.00	129	0.00*	0.11	1.00	62	0.33	0.08	1.00	56	0.13
Ti	s	0.663	0.00*	0.04	0.00*	1.506	0.00*	0.07	1.00	1.744	0.03*	0.07	1.00	1.939	0.10
Te	s	1.053	0.00*	0.12	1.00	1.396	0.00*	0.10	1.00	2.905	1.00	0.12	1.00	2.760	0.11
**COUGH**															
ELM	spk/s	302	0.00*	0.33	0.04*	521	0.00*	0.22	0.86	55	1.00	0.07	1.00	55	0.09
ILM	spk/s	238	0.00*	0.11	1.00	154	0.00*	0.14	1.00	37	1.00	0.10	1.00	36	0.10
LUMBAR	spk/s	235	0.00*	0.07	1.00	536	0.00*	0.10	1.00	76	1.00	0.11	1.00	76	0.06
PHRENIC	spk/s	348	0.00*	0.14	0.16	705	0.00*	0.16	0.07	98	1.00	0.04	1.00	97	0.05
Ti	s	0.494	0.00*	0.20	1.00	0.503	0.00*	0.23	1.00	2.471	1.00	0.09	1.00	2.302	0.12
Te	s	0.564	0.00*	0.24	1.00	0.476	0.00*	0.19	1.00	3.490	1.00	0.19	1.00	3.242	0.15

*The behaviors of the networks in Rybak et al. (2008) and Poliaček et al. (2011) and a model without recently added speculative connections are compared with the model in this paper. The mean of the peak (in each respiratory cycle) firing rates for four motor populations and the mean inspiratory (Ti) and expiratory (Te) phase durations are shown. Each mean for each previous model is followed by the *p*-value from a two-sided *t*-test with non-pooled SD for the difference in means between the previous model and the current model. The cycle counts for Rybak, Poliaček, No speculative, and Current, respectively, were 67, 40, 24, and 24 for eupnea and 56, 60, 10, and 10 for cough. The table also shows the coefficient of variation (CV) for each firing rate or phase duration for assessment of differences in variability between the models. Each CV is followed by the *p*-value from an *F*-test for the ratio of variances of the observations divided by their mean between the previous model and the current model. *p*-Values have been adjusted for multiple testing (Holm, 1979); significant values (at the 0.05 level) are marked with an asterisk*.

**Figure 8:**
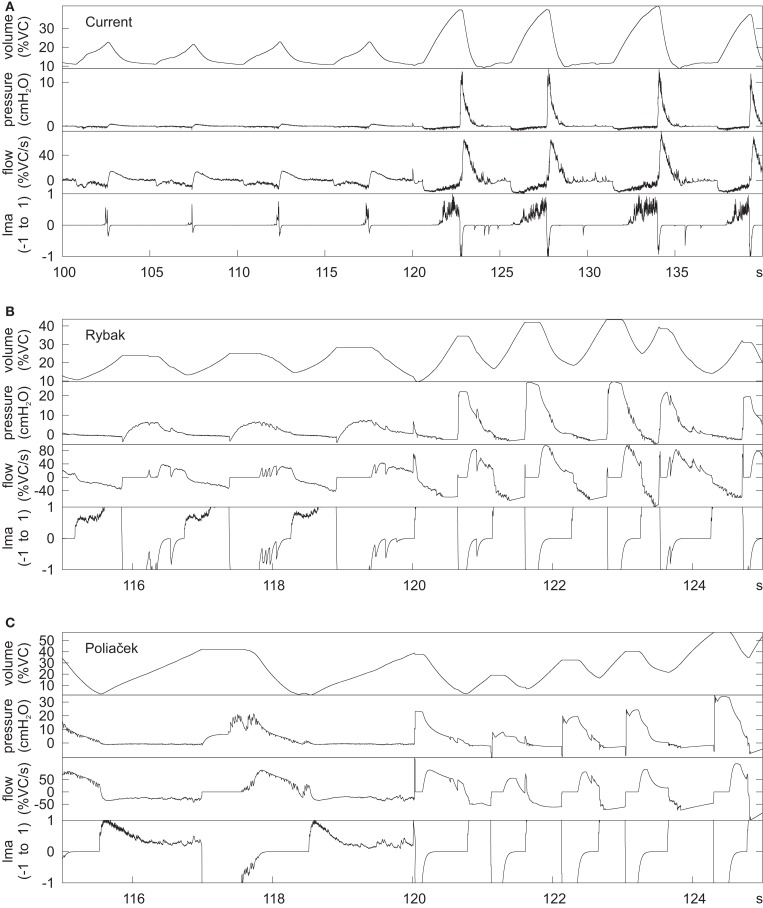
**Outputs of the mechanical model when linked to the current network model (A) and the networks in (B) (Rybak et al., 2008) and (C) (Poliaček et al., 2011)**. The earlier networks were designed without a mechanical model, but were connected to the current mechanical model to generate these plots. The plots are lung volume, alveolar pressure, tracheal flow, and laryngeal muscle activation (lma). The value of lma is 1 for a maximally open glottis, 0 for the resting diameter, and −1 for a closed glottis. The first few cycles are eupneic cycles, and the rest are coughs. The time scale is different for the current model because it was designed with slower cycles to match human respiration.

These antecedent variants of the present neuronal network model were designed without a linked biomechanical system. The new joint neuromechanical model aids tuning of phase-timing relationships and the scaling of model motor outputs. To illustrate this model feature, we linked the earlier network models to the new biomechanical model. We note that the phrenic and lumbar motor neuron activities from the Rybak and Poliaček models are not strictly comparable because the current model has a second population of each type of motor neuron to model recruitment with increased drive. Lung stretch receptor inputs to the previous network models remained filtered versions of the phrenic motor output (i.e., there was no feedback from the mechanical models to the network). Figure 8 shows records of lung volume, alveolar pressure, tracheal flow, and laryngeal muscle activation from the current neuromechanical model (Figure 8A) and for the two earlier models when connected to the biomechanical system (Figures 8B,C). The scaling and activation of the laryngeal muscles caused airway closure prior to each eupneic expiration when using the older models’ outputs (lma = −1, flow flattens at 0). During cough in the previous models, the next inspiration started before the previous expiration was complete, resulting in a progressive increase in lung volume from cough to cough.

#### Influence of some added network connections

As noted in Sections “Linking the Neural Network and Biomechanical Models” and “Additional Enhancements to the Current Network Model,” the current network model includes lung afferents responsive to lung deflation and presynaptic inhibition by E-Dec-Tonic neurons of excitatory inputs from the I-Aug population to I-Dec_2 neurons. The latter feature was added to prolong I-Dec_2 neuron activity when E-Dec-Tonic neuron I-phase activity is reduced. Removal of these three speculative model elements resulted in shorter inspiratory phase durations (Table 5, “No speculative” and “Current” columns).

## Discussion

The new biomechanical model system detailed in the Results incorporates several features developed using measures from human subjects. These include a model of the abdominal volume that captures the interaction of the diaphragm, rib cage, and abdominal wall based on Grassino et al. (1978), an abdominal wall model based on measurements of the curvature of the abdomen by Song et al. (2006) taken during insufflation for laparoscopic surgery, and a model of the larynx using results from Tully et al. (1990, 1991). The mechanical model was linked to an enhanced version of a previously described computational network model (Rybak et al., 2008; Poliaček et al., 2011) with IF neuron populations, connections, and other properties measured or inferred from *in vivo* and *in vitro* studies of mammalian brainstem circuits for breathing and cough (Shannon et al., 2000; Segers et al., 2008; Lindsey et al., 2012).

The joint neuromechanical system is a prototype for study of the neural control of breathing and airway defensive behaviors. To our knowledge, computational neural network models of cough generation have been evaluated previously using measures of motor neuron burst sequences, phase durations, and the time varying firing rates of neuron populations that only indirectly reflect possible muscle activation patterns and their attendant biomechanical consequences. The new model generated eupneic breathing and cough motor patterns together with corresponding alterations in lung volume, tracheal air flow, and other relevant metrics of cough mechanics. The present results also show the utility of the model as an aid for tuning the motor pattern and as a tool to evaluate the efficacy of phase-timing relationships.

A specific goal of this project was to assess model output during cough under conditions of altered inspiratory drive. We were motivated in part by the recent observation that lung operating volume at the onset of the compressive phase of cough influences subsequent air flow velocities during the expulsive phase (Smith et al., 2012). Inspiratory drive was altered by two distinct approaches: (i) increased gain of phrenic motor neuron activation of the diaphragm, and (ii) sequential modulation or deletion of synaptic inputs to inspiratory premotor populations. Both perturbations altered cough inspiratory volume. We also found changes in expulsive phase air flow associated with corresponding changes in peak abdominal pressure attributable to cough mechanics, results that could not have been achieved by measures of the motor pattern output alone. In the first protocol, higher end inspiratory volumes resulted in greater tracheal air flow during the subsequent expulsive phase under the same abdominal expiratory motor drives. Under the second protocol, the difference in operating volumes between the enhanced drive and reduced excitatory drive states was associated with corresponding reductions in expiratory flow and peak abdominal pressure.

### Discrepancies with experimental results and model limitations

The discrepancy between the present results and those of Smith et al. (2012) is noteworthy. The latter study found changes in expulsive flow rates during voluntary coughs from different operating volumes in the absence of significant alterations in thoracic or abdominal pressures, whereas we found changes in flow associated with changes in abdominal pressure, despite no change in abdominal drive. The change in expiratory pressure in the model is due to the action of the intercostal and accessory muscles; the expiratory pressure increases because the pressure from those muscles in the model increases with rib cage volume at constant activation. Our model calculates the expiratory pressure generated by the intercostal and accessory muscles at TLC and full abdominal activation necessary to produce maximal expiratory pressure, and at RV to complete the pressure balance on the rib cage (a much smaller number; see “Additional Enhancements to the Current Network Model”). The model assumes that the expiratory pressure generated by the intercostal and accessory muscles scales linearly with rib cage volume between RV and TLC, and linearly with abdominal activation, leading to higher pressures at higher rib cage volumes with equal activation. The experimentally observed increase in maximal expiratory pressure with rib cage volume could be due to increased activation of the intercostals or improved mechanical advantage at larger volumes or a combination of the two. If improved mechanical advantage is a factor, the brainstem would have to reduce drive at higher volumes during cough to avoid higher pressures, suggesting that it may be sensing the generated pressures and adjusting drive accordingly (see e.g., discussion in “Tonic Expiratory Neurons: Model Results and Predictions”). A refined configuration to accommodate separate intercostal muscles, intercostal motor neuron populations, and muscle afferents (Shannon, 1986) and their control of the chest wall would be useful in this regard.

When we ran the biomechanical model in isolation with reduced abdominal drive at higher volumes in order to maintain an equal peak pressure (see Figure 7), we saw peak flow rate changes comparable in magnitude to those seen by Smith et al. (2012, Figure 3B), due to the increasing recoil pressure of the lung at higher volumes. The peak flow rates were comparable despite the fact that the peak abdominal pressure in our simulation was less than half that observed by Smith et al. This lower resistance is likely due to the fact that we did not model airway collapse, which is known to limit peak flow rates (Knudson et al., 1974).

We found that increased flow during cough at higher lung volume is primarily due to increased lung recoil pressure. The lung recoil pressure certainly increases with lung volume, but the accuracy of the resulting flow in the model may be affected by certain known limitations of the model: (i) airway collapse during cough is not modeled, resulting in an underestimate of airway resistance; (ii) the lung compliance is assumed to be constant in the model, whereas it is thought to vary with lung volume *in vivo*; (iii) the model does not take into account hysteresis in the lung flow-volume curve; (iv) volume changes due to blood shift out of the trunk during cough are not modeled; and (v) volume changes due to gas compression are not modeled (see Smith et al., 2012 for data on volume changes due to blood shift and gas compression). Nevertheless, our model suggests the hypothesis that the increased flow during cough is primarily due to increased lung recoil pressure.

### Tonic expiratory neurons: Model results and predictions

The model incorporated multiple target sites for cough drive modulation, a feature of the network architecture based on correlational linkage maps of functional connectivity and associated neuronal responses to stimuli that either enhance or suppress inspiratory drive *in vivo* (Lindsey et al., 1998; Shannon et al., 2000; Poliaček et al., 2011; Ott et al., 2012). Deletion of excitatory mechanisms for cough inspiratory drive resulted in reductions in peak lung volume and a subsequently diminished peak air flow relative to baseline during the expulsive phase (Figure 6B). Although removal of the disinhibitory component of the drive enhancement mechanism mediated by the E-Dec-Tonic population did not further reduce expulsive phase air flow velocity, it did lead to both an additional decrease in inspiratory phase lung operating volume and a reduced expulsive phase peak abdominal pressure relative to baseline values, even though peak abdominal drive did not change.

We have previously proposed the hypothesis that tonic expiratory neurons provide a reservoir for inspiratory drive modulation. Suppression of their inspiratory phase activity during central chemoreceptor-mediated drive and spike train cross-correlation analyses both suggest that VRC tonic E neuron inhibition of premotor inspiratory neurons is reduced in high drive states, at least in part, by increased I-Dec neuron inhibition (Ott et al., 2012).

The present model included a network “module” previously introduced (Poliaček et al., 2011) for baroreceptor modulation of breathing. That circuit, inferred from spike train correlational linkages and neuron responses to baroreceptor stimulation (Lindsey et al., 1998), operated via excitatory and disinhibitory raphé neuron influences acting upon VRC E-Dec-Phasic and E-Dec-Tonic neuron populations. Simulations of baroreceptor activation using prior models (Poliaček et al., 2011; Lindsey et al., 2012) with circuits inferred from *in vivo* observations (see references in Lindsey et al., 1998; Poliaček et al., 2011; Ott et al., 2012) generated prolongation of expiration and reduced inspiratory drive during both eupneic respiratory cycles and evoked cough.

Collectively, these data support the hypothesis that inhibition of the E-Dec-Tonic population in the cough network amplifies inspiratory drive via disinhibition. Experimental data consistent with this hypothesis is presented in a companion paper (Segers et al., 2012). Modulation of tonic expiratory neuron activity could also operate in a push-pull mechanism in which cough drive is balanced against the potentially suppressive influences of blood pressure changes caused by cough mechanics.

### Future directions

The present model provides a framework for integrating respiratory network model development with respiratory mechanics and will guide and facilitate scaling and timing of motor neuron activity patterns and functionally antecedent connectivity for the generation of breathing, cough, and swallow. The simulations of cough and breathing suggest that an important area of focus for future modeling efforts will be reconciliation of known differential effects of pulmonary volume-related feedback on breathing and airway defensive behaviors such as coughing and the expiratory reflex. Specific components of the model that are proposed to have the greatest effect on its potential for prediction are the gain of pulmonary volume-related feedback and the interaction of this feedback with cough-related sensory information. Future models should also guide experiments targeting the control of behavior that must be tightly coordinated with breathing, such as sniffing, swallowing, and vocalization.

## Conflict of Interest Statement

The authors declare that the research was conducted in the absence of any commercial or financial relationships that could be construed as a potential conflict of interest.

## References

[B1] A.D.A.M. Medical Encyclopedia (2012). Rapid Shallow Breathing. Available at: http://www.nlm.nih.gov/medlineplus/ency/article/007198.htm [accessed May 9, 2012].

[B2] Agostoni E. Gurtner G. Torri G. Rahn H. (1966). Respiratory mechanics during submersion and negative-pressure breathing. J. Appl. Physiol. 21, 251–258 5903920 10.1152/jappl.1966.21.1.251

[B3] Agostoni E. Rahn H. (1960). Abdominal and thoracic pressures at different lung volumes. J. Appl. Physiol. 15, 1087–1092 13681667 10.1152/jappl.1960.15.6.1087

[B4] Artemiadis P. K. Kyriakopoulos K. J. (2005). “Teleoperation of a robot manipulator using EMG signals and a position tracker,” in IEEE/RSJ International Conference on Intelligent Robots and Systems, Edmonton, 2005

[B5] Baekey D. M. Morris K. F. Nuding S. C. Segers L. S. Li Z. Lindsey B. G. Shannon R. (2001). Involvement of ventral respiratory group neurons in the fictive expiration reflex. FASEB J. abstr. 15, A798

[B6] Baier H. Wanner A. Zarzecki S. Sackner M. A. (1977). Relationships among glottis opening, respiratory flow, and upper airway resistance in humans. J. Appl. Physiol. 43, 603–611 908674 10.1152/jappl.1977.43.4.603

[B7] Barnas G. M. Yoshino K. Stamenovic D. Kikuchi Y. Loring S. H. Mead J. (1989). Chest wall impedance partitioned into rib cage and diaphragm-abdominal pathways. J. Appl. Physiol. 66, 350–359 10.1063/1.343880 2917941

[B8] Bergren D. R. Peterson D. F. (1993). Identification of vagal sensory receptors in the rat lung: are there subtypes of slowly adapting receptors? J. Physiol. 464, 681–698 8229824 10.1113/jphysiol.1993.sp019657 PMC1175408

[B9] Bolser D. C. Davenport P. W. Golder F. J. Baekey D. M. Morris K. F. Lindsey B. G. Shannon R. (2003). “Neurogenesis of cough,” in Cough: Causes, Mechanisms and Therapy, 1st Edn, eds Boushey H. Chung K. Widdicombe J. G. (Malden: Blackwell Publishing Ltd.), 173–180

[B10] Brancatisano T. Collett P. W. Engel L. A. (1983). Respiratory movements of the vocal cords. J. Appl. Physiol. 54, 1269–1276 6863086 10.1152/jappl.1983.54.5.1269

[B11] Braun N. M. Arora N. S. Rochester D. F. (1982). Force-length relationship of the normal human diaphragm. J. Appl. Physiol. 53, 405–412 7118662 10.1152/jappl.1982.53.2.405

[B12] Breen B. J. Gerken W. C. Butera R. J. (2003). Hybrid integrate-and-fire model of a bursting neuron. Neural Comput. 15, 2843–2862 10.1162/089976603322518768 14629870

[B13] Cheng L. Ivanova O. Fan H.-H. Khoo M. C. K. (2010). An integrative model of respiratory and cardiovascular control in sleep-disordered breathing. Respir. Physiol. Neurobiol. 174, 4–28 10.1016/j.resp.2010.06.001 20542148 PMC2965826

[B14] Cheng L. Khoo M. C. (2012). Modeling the autonomic and metabolic effects of obstructive sleep apnea: a simulation study. Front. Physiol. 2:111 10.3389/fphys.2011.00111 22291654 PMC3250672

[B15] Chiel H. J. Ting L. H. Ekeberg Ö. Hartmann M. J. Z. (2009). The brain in its body: motor control and sensing in a biomechanical context. J. Neurosci. 29, 12807–12814 10.1523/JNEUROSCI.3338-09.2009 19828793 PMC2794418

[B16] Chow J. W. Darling W. G. (1999). The maximum shortening velocity of muscle should be scaled with activation. J. Appl. Physiol. 86, 1025–1031 10066719 10.1152/jappl.1999.86.3.1025

[B17] Cluzel P. Similowski T. Chartrand-Lefebvre C. Zelter M. Derenne J.-P. Grenier P. A. (2000). Diaphragm and chest wall: assessment of the inspiratory pump with MR imaging – preliminary observations. Radiology 215, 574–583 10796942 10.1148/radiology.215.2.r00ma28574

[B18] De Troyer A. Estenne M. Ninane V. Van Gansbeke D. Gorini M. (1990). Transversus abdominis muscle function in humans. J. Appl. Physiol. 68, 1010–1016 2140344 10.1152/jappl.1990.68.3.1010

[B19] Dick T. E. Shannon R. Lindsey B. G. Nuding S. C. Segers L. S. Baekey D. M. Morris K. F. (2008). Pontine respiratory-modulated activity before and after vagotomy in decerebrate cats. J. Physiol. 586, 4265–4282 10.1113/jphysiol.2008.152108 18599543 PMC2652175

[B20] D’Urzo A. D. Rubinstein I. Lawson V. G. Vassal K. P. Rebuck A. S. Slutsky A. S. Hoffstein V. (1988). Comparison of glottic areas measured by acoustic reflections vs. computerized tomography. J. Appl. Physiol. 64, 367–370 3356656 10.1152/jappl.1988.64.1.367

[B21] Estenne M. Yernault J. C. De Troyer A. (1985). Rib cage and diaphragm-abdomen compliance in humans: effects of age and posture. J. Appl. Physiol. 59, 1842–1848 4077793 10.1152/jappl.1985.59.6.1842

[B22] Fitz-Clarke J. R. (2007). Computer simulation of human breath-hold diving: cardiovascular adjustments. Eur. J. Appl. Physiol. Occup. Physiol. 100, 207–224 10.1007/s00421-007-0421-z 17323072

[B23] Gaumann A. Hoeckel M. Konerding M. (1999). The anatomic basis of the transversus and rectus abdominis musculoperitoneal (TRAMP) composite flap. Hernia 3, 39–41 10.1007/BF01576742

[B24] Gilroy R. J. Lavietes M. H. Loring S. H. Mangura B. T. Mead J. (1985). Respiratory mechanical effects of abdominal distension. J. Appl. Physiol. 58, 1997–2003 3159715 10.1152/jappl.1985.58.6.1997

[B25] Goldman M. D. Grassino A. Mead J. Sears T. A. (1978). Mechanics of the human diaphragm during voluntary contraction: dynamics. J. Appl. Physiol. 44, 840–848 670007 10.1152/jappl.1978.44.6.840

[B26] Grassino A. Goldman M. D. Mead J. Sears T. A. (1978). Mechanics of the human diaphragm during voluntary contraction: statics. J. Appl. Physiol. 44, 829–839 149776 10.1152/jappl.1978.44.6.829

[B27] Grimby G. Goldman M. Mead J. (1976). Respiratory muscle action inferred from rib cage and abdominal V-P partitioning. J. Appl. Physiol. 41, 739–751 993162 10.1152/jappl.1976.41.5.739

[B28] Harris R. S. (2005). Pressure-volume curves of the respiratory system. Respir. Care 50, 78–98 15636647

[B29] Hatze H. (1981). Myocybernetic Control Models of Skeletal Muscle: Characteristics and Applications. Pretoria: University of South Africa

[B30] Hey E. N. Price J. F. (1982). Nasal conductance and effective airway diameter. J. Physiol. (Lond.) 330, 429–437 7175748 10.1113/jphysiol.1982.sp014349 PMC1225306

[B31] Hill A. V. (1938). The heat of shortening and the dynamic constants of muscle. Proc. R. Soc. Lond. B Biol. Sci. 126, 136–195 10.1098/rspb.1938.0050 18152150

[B32] Holm S. (1979). A simple sequentially rejective multiple test procedure. Scand. J. Statist. 6, 65–70

[B33] Hutchison A. A. Lindsey B. G. (2009). “Modeling central brainstem mechanisms in eupnea, grunting, incremental breathing and vagotomy,” in American Journal of Respiratory and Critical Care Medicine (Abstract) from American Thoracic Society International Conference, San Diego, CA

[B34] Iscoe S. D. (1998). Control of abdominal muscles. Prog. Neurobiol. 56, 433–506 10.1016/S0301-0082(98)00046-X 9775401

[B35] Iscoe S. D. Gordon S. P. (1992). Chest wall distortion and discharge of pulmonary slowly adapting receptors. J. Appl. Physiol. 73, 1619–1625 1447113 10.1152/jappl.1992.73.4.1619

[B36] Kamel K. S. Lau G. Stringer M. D. (2009). *In vivo* and *in vitro* morphometry of the human trachea. Clin. Anat. 22, 571–579 10.1002/ca.20841 19544298

[B37] Kim M. J. Druz W. S. Danon J. Machnach W. Sharp J. T. (1976). Mechanics of the canine diaphragm. J. Appl. Physiol. 41, 369–382 965306 10.1152/jappl.1976.41.3.369

[B38] Knudson R. J. Mead J. Knudson D. E. (1974). Contribution of airway collapse to supramaximal expiratory flows. J. Appl. Physiol. 36, 653–667 4829903 10.1152/jappl.1974.36.6.653

[B39] Konno K. Mead J. (1967). Measurement of the separate volume changes of rib cage and abdomen during breathing. J. Appl. Physiol. 22, 407–422 4225383 10.1152/jappl.1967.22.3.407

[B40] Kreith F. Berger S. Churchill S. Tullis J. P. Tullis B. White F. Kumar A. Todd J. Chen J. Irvine T. Capobianchi M. Kennedy F. Booser E. R. Wilcock D. Boehm R. Reitz R. Kim J. McDonald A. Sherif S. Bhushan B. (2004). “Fluid mechanics,” in The CRC Handbook of Mechanical Engineering, 2nd Edn, eds Kreith F. Goswami D. Y. (Boca Raton: CRC Press), 3.1–3.232

[B41] Laplace P. S. (1808). Traité de mécanique céleste: supplément au dixième livre du traité de méchanique céleste sur l’action capillaire. Paris: Duprat

[B42] Lichtenstein O. Ben-Haim S. A. Saidel G. M. Dinnar U. (1992). Role of the diaphragm in chest wall mechanics. J. Appl. Physiol. 72, 568–574 1559934 10.1152/jappl.1992.72.2.568

[B43] Lindsey B. G. Arata A. Morris K. F. Hernandez Y. M. Shannon R. (1998). Medullary raphé neurones and baroreceptor modulation of the respiratory motor pattern in the cat. J. Physiol. 512, 863–882 10.1111/j.1469-7793.1998.863bd.x 9769428 PMC2231246

[B44] Lindsey B. G. Rybak I. A. Smith J. C. (2012). Computational models and emergent properties of respiratory neural networks. Compr. Physiol. 2, 1619–1670 10.1002/cphy.c110016 PMC3656479 23687564

[B45] Loring S. H. Mead J. (1982). Action of the diaphragm on the rib cage inferred from a force-balance analysis. J. Appl. Physiol. 53, 756–760 10.1063/1.329944 6215388

[B46] Luck J. C. (1970). Afferent vagal fibres with an expiratory discharge in the rabbit. J. Physiol. (Lond.) 211, 63–71 5500999 10.1113/jphysiol.1970.sp009266 PMC1395593

[B47] MacGregor R. J. (1987). Neural and Brain Modeling. New York: Academic Press

[B48] Macklem P. T. Gross D. Grassino G. A. Roussos C. (1978). Partitioning of inspiratory pressure swings between diaphragm and intercostal/accessory muscles. J. Appl. Physiol. 44, 200–208 632159 10.1152/jappl.1978.44.2.200

[B49] Mantilla C. B. Sieck G. C. (2011). Phrenic motor unit recruitment during ventilatory and non-ventilatory behaviors. Respir. Physiol. Neurobiol. 179, 57–63 10.1016/j.resp.2011.06.028 21763470 PMC3183333

[B50] Matsumoto S. Ikeda M. Nishikawa T. Yoshida S. Tanimoto T. Ito M. Saiki C. Takeda M. (2002). Excitatory mechanism of deflationary slowly adapting pulmonary stretch receptors in the rat lung. J. Pharmacol. Exp. Ther. 300, 597–604 10.1124/jpet.300.2.668 11805222

[B51] McCool F. D. (2006). Global physiology and pathophysiology of cough. Chest 129, 48S–53S 10.1378/chest.129.1_suppl.48S 16428691

[B52] Mead J. Loring S. H. (1982). Analysis of volume displacement and length changes of the diaphragm during breathing. J. Appl. Physiol. 53, 750–755 6215387 10.1152/jappl.1982.53.3.750

[B53] Molkov Y. I. Abdala A. P. L. Bacak B. J. Smith J. C. Paton J. F. R. Rybak I. A. (2010). Late-expiratory activity: emergence and interactions with the respiratory CPG. J. Neurophysiol. 104, 2713–2729 10.1152/jn.00334.2010 20884764 PMC2997033

[B54] Nail B. S. Sterling G. M. Widdicombe J. G. (1972). Patterns of spontaneous and reflexly-induced activity in phrenic and intercostal motoneurons. Exp. Brain Res. 15, 318–332 10.1007/BF00235915 5070223

[B55] Ott M. M. Nuding S. C. Segers L. S. O’Connor R. Morris K. F. Lindsey B. G. (2012). Central chemoreceptor modulation of breathing via multipath tuning in medullary ventrolateral respiratory column circuits. J. Neurophysiol. 107, 603–617 10.1152/jn.00808.2011 21994272 PMC3349622

[B56] Paintal A. S. (1955). Impulses in vagal afferent fibres from specific pulmonary deflation receptors: the response of these receptors to phenyl diguanide, potato starch, 5-hydroxytryptamine and nicotine, and their rôle in respiratory and cardiovascular reflexes. Exp. Physiol. 40, 89–111 10.1113/expphysiol.1955.sp001116 14371992

[B57] Permutt S. Martin H. B. (1960). Static pressure-volume characteristics of lungs in normal males. J. Appl. Physiol. 15, 819–825 13734448 10.1152/jappl.1960.15.5.819

[B58] Poliaček I. Morris K. F. Lindsey B. G. Segers L. S. Rose M. J. Corrie L. W.-C. Wang C. Pitts T. E. Davenport P. W. Bolser D. C. (2011). Blood pressure changes alter tracheobronchial cough: computational model of the respiratory-cough network and in vivo experiments in anesthetized cats. J. Appl. Physiol. 111, 861–873 10.1152/japplphysiol.00458.2011 21719729 PMC3174787

[B59] Ratnovsky A. Elad D. Halpern P. (2008). Mechanics of respiratory muscles. Respir. Physiol. Neurobiol. 163, 82–89 10.1016/j.resp.2008.04.019 18583200

[B60] Ratnovsky A. Zaretsky U. Shiner R. J. Elad D. (2003). Integrated approach for in vivo evaluation of respiratory muscles mechanics. J. Biomech. 36, 1771–1784 10.1016/S0021-9290(03)00232-X 14614931

[B61] Reid M. B. Feldman H. A. Miller M. J. (1987). Isometric contractile properties of diaphragm strips from alcoholic rats. J. Appl. Physiol. 63, 1156–1164 3654462 10.1152/jappl.1987.63.3.1156

[B62] Renotte C. Remy M. Saucez P. (1998). Dynamic model of airway pressure drop. Med. Biol. Eng. Comput. 36, 101–106 10.1007/BF02522865 9614756

[B63] Riddle W. Younes M. (1981). A model for the relation between respiratory neural and mechanical outputs. II. Methods. J. Appl. Physiol. 51, 979–989 7298441 10.1152/jappl.1981.51.4.979

[B64] Roca J. Burgos F. Sunyer J. Saez M. Chinn S. Anto J. M. Rodriguez-Roisin R. Quanjer P. H. Nowak D. Burney P. (1998). References values for forced spirometry. Group of the European community respiratory health survey. Eur. Respir. J. 11, 1354–1362 10.1183/09031936.98.11061354 9657579

[B65] Rohrer F. (1915). Der stromungswiderstand in den menschlichen atemwegen und der einfluss der unregelmassigen verzweigung des bronchialsystems auf den atmungsverlauf in verschiedenen lungenbezirken. Archiv fur die Gesamte Physiologie des Menschen und der Tiere 162, 225–299

[B66] Rosen J. Fuchs M. B. Arcan M. (1999). Performances of Hill-type and neural network muscle models: toward a myosignal-based exoskeleton. Comput. Biomed. Res. 32, 415–439 10.1006/cbmr.1999.1524 10529300

[B67] Rybak I. A. O’Connor R. Ross A. Shevtsova N. A. Nuding S. C. Segers L. S. Shannon R. Dick T. E. Dunin-Barkowski W. L. Orem J. M. Solomon I. C. Morris K. F. Lindsey B. G. (2008). Reconfiguration of the pontomedullary respiratory network: a computational modeling study with coordinated in vivo experiments. J. Neurophysiol. 100, 1770–1799 10.1152/jn.90416.2008 18650310 PMC2576193

[B68] Segers L. S. Nuding S. C. Dick T. E. Shannon R. Baekey D. M. Solomon I. C. Morris K. F. Lindsey B. G. (2008). Functional connectivity in the pontomedullary respiratory network. J. Neurophysiol. 100, 1749–1769 10.1152/jn.90414.2008 18632881 PMC2576196

[B69] Segers L. S. Nuding S. C. Vovk A. Baekey D. M. O’Connor R. Morris K. F. Lindsey B. G. Shannon R. Bolser D. C. (2012). Discharge identity of medullary inspiratory neurons is altered during repetitive fictive cough. Front. Physiol. 3:223 10.3389/fphys.2012.00223 22754536 PMC3386566

[B70] Shannon R. (1986). “Reflexes from the respiratory muscles and controvertebral joints,” in Handbook of Physiology. The Respiratory System. Control of Breathing, Sect. 3, Part 1, eds Cherniack N. S. Widdicombe J. G. (Washington, DC: American Physiological Society), 431–447

[B71] Shannon R. Baekey D. M. Morris K. F. Li Z. Lindsey B. G. (2000). Functional connectivity among ventrolateral medullary respiratory neurones and responses during fictive cough in the cat. J. Physiol. 525, 207–224 10.1111/j.1469-7793.2000.00207.x 10811738 PMC2269920

[B72] Shannon R. Baekey D. M. Morris K. F. Lindsey B. G. (1998). Ventrolateral medullary respiratory network and a model of cough motor pattern generation. J. Appl. Physiol. 84, 2020–2035 9609797 10.1152/jappl.1998.84.6.2020

[B73] Shannon R. Baekey D. M. Morris K. F. Nuding S. C. Segers L. S. Lindsey B. G. (2004). Production of reflex cough by brainstem respiratory networks. Pulm. Pharmacol. Ther. 17, 369–376 10.1016/j.pupt.2004.09.022 15564078

[B74] Sharpey-Schafer E. P. (1953). Effects of coughing on intra-thoracic pressure, arterial pressure and peripheral blood flow. J. Physiol. (Lond.) 122, 351–357 13118544 10.1113/jphysiol.1953.sp005004 PMC1366120

[B75] Siafakas N. M. Morris A. J. Green M. (1979). Thoracoabdominal mechanics during relaxed and forced vital capacity. J. Appl. Physiol. 47, 38–42 468671 10.1152/jappl.1979.47.1.38

[B76] Simpson L. L. (1968). Sizing piping for process plants. Chem. Eng. 75, 192–214

[B77] Smith J. Bellemare F. (1987). Effect of lung volume on *in vivo* contraction characteristics of human diaphragm. J. Appl. Physiol. 62, 1893–1900 3597263 10.1152/jappl.1987.62.5.1893

[B78] Smith J. A. Aliverti A. Quaranta M. McGuinness K. Kelsall A. Earis J. Calverley P. M. (2012). Chest wall dynamics during voluntary and induced cough in healthy volunteers. J. Physiol. (Lond.) 590, 563–574 22144580 10.1113/jphysiol.2011.213157 PMC3379701

[B79] Song C. Alijani A. Frank T. Hanna G. B. Cuschieri A. (2006). Mechanical properties of the human abdominal wall measured in vivo during insufflation for laparoscopic surgery. Surg. Endosc. 20, 987–990 10.1007/s00464-005-0676-6 16738998

[B80] Suárez A. A. Pessolano F. A. Monteiro S. G. Ferreyra G. Capria M. E. Mesa L. Dubrovsky A. De Vito E. L. (2002). Peak flow and peak cough flow in the evaluation of expiratory muscle weakness and bulbar impairment in patients with neuromuscular disease. Am. J. Phys. Med. Rehabil. 81, 506–511 10.1097/00002060-200207000-00007 12131177

[B81] Tully A. Brancatisano A. Loring S. H. Engel L. A. (1990). Relationship between thyroarytenoid activity and laryngeal resistance. J. Appl. Physiol. 68, 1988–1996 2361900 10.1152/jappl.1990.68.5.1988

[B82] Tully A. Brancatisano A. Loring S. H. Engel L. A. (1991). Influence of posterior cricoarytenoid muscle activity on pressure-flow relationship of the larynx. J. Appl. Physiol. 70, 2252–2258 1864806 10.1152/jappl.1991.70.5.2252

[B83] Wei J. Y. Shen E. (1985). Vagal expiratory afferent discharges during spontaneous breathing. Brain Res. 335, 213–219 10.1016/0006-8993(85)90472-X 3924341

[B84] Younes M. Riddle W. (1981). A model for the relation between respiratory neural and mechanical outputs. I. Theory. J. Appl. Physiol. 51, 963–978 7298440 10.1152/jappl.1981.51.4.963

[B85] Younes M. Riddle W. Polacheck J. (1981). A model for the relation between respiratory neural and mechanical outputs. III Validation . J. Appl. Physiol. 51, 990–1001 7298442 10.1152/jappl.1981.51.4.990

[B86] Yu J. Wang Y. F. Zhang J. W. (2003). Structure of slowly adapting pulmonary stretch receptors in the lung periphery. J. Appl. Physiol. 95, 385–393 12665534 10.1152/japplphysiol.00137.2003

